# Monitoring the interplay between transposable element families and DNA methylation in maize

**DOI:** 10.1371/journal.pgen.1008291

**Published:** 2019-09-09

**Authors:** Jaclyn M. Noshay, Sarah N. Anderson, Peng Zhou, Lexiang Ji, William Ricci, Zefu Lu, Michelle C. Stitzer, Peter A. Crisp, Candice N. Hirsch, Xiaoyu Zhang, Robert J. Schmitz, Nathan M. Springer

**Affiliations:** 1 Department of Plant and Microbial Biology, University of Minnesota, St. Paul MN, United States of America; 2 Institute of Bioinformatics, University of Georgia, Athens GA, United States of America; 3 Department of Plant Biology, University of Georgia, Athens GA, United States of America; 4 Department of Genetics, University of Georgia, Athens GA, United States of America; 5 Department of Plant Sciences, University of California Davis, Davis CA, United States of America; 6 Department of Agronomy and Plant Genetics, University of Minnesota, St. Paul MN, United States of America; University of Lille, FRANCE

## Abstract

DNA methylation and epigenetic silencing play important roles in the regulation of transposable elements (TEs) in many eukaryotic genomes. A majority of the maize genome is derived from TEs that can be classified into different orders and families based on their mechanism of transposition and sequence similarity, respectively. TEs themselves are highly methylated and it can be tempting to view them as a single uniform group. However, the analysis of DNA methylation profiles in flanking regions provides evidence for distinct groups of chromatin properties at different TE families. These differences among TE families are reproducible in different tissues and different inbred lines. TE families with varying levels of DNA methylation in flanking regions also show distinct patterns of chromatin accessibility and modifications within the TEs. The differences in the patterns of DNA methylation flanking TE families arise from a combination of non-random insertion preferences of TE families, changes in DNA methylation triggered by the insertion of the TE and subsequent selection pressure. A set of nearly 70,000 TE polymorphisms among four assembled maize genomes were used to monitor the level of DNA methylation at haplotypes with and without the TE insertions. In many cases, TE families with high levels of DNA methylation in flanking sequence are enriched for insertions into highly methylated regions. The majority of the >2,500 TE insertions into unmethylated regions result in changes in DNA methylation in haplotypes with the TE, suggesting the widespread potential for TE insertions to condition altered methylation in conserved regions of the genome. This study highlights the interplay between TEs and the methylome of a major crop species.

## Author summary

Maize, like many other crops, has a complex genome that is primarily composed of transposable elements (TEs). These TEs play major roles in shaping the epigenome and have the potential to influence genes or regulatory regions. Although many studies assess the average patterns of chromatin at all TEs, there is evidence for significant variation among different TE families. We find that TE families have distinct patterns of DNA methylation in flanking regions. These differences can be explained by a combination of preferential insertion of some families into methylated or unmethylated genomic regions as well as the potential for some TE families to trigger methylation of flanking regions. Understanding the interplay of TE families and chromatin can help understand the mechanisms through which TEs influence genes and shape crop genomes.

## Introduction

Highly repetitive regions, mostly derived from transposable elements (TEs), account for the majority of DNA sequence in most crop genomes. TEs include several different types of elements [1]. Class I TEs, also known as retrotransposons, utilize an RNA intermediates and many have long terminal repeats (LTRs). Class II TEs include DNA transposons and helitrons and utilize DNA intermediates. The DNA transposons are flanked by terminal inverted repeats (TIRs). The *Zea mays* B73v4 genome contains ~300,000 structurally intact TEs (defined based on presence of structural features such as TIR/LTR and target site duplication (TSD) belonging to ~26,000 families [2], providing opportunities to understand how TEs interact with chromatin. There are high abundance families as well as many smaller families of TEs [2,3], with family sizes ranging from 1 to >15,000 in the B73v4 reference genome. These structurally intact maize TEs account for ~65% of the maize genome [2] and estimates based on repeat masking of the maize genome, which are more sensitive for detecting fragments of TEs, suggest that >80% of the maize genome is derived from TEs [4]. Unlike the model plant species *Arabidopsis thaliana*, TEs in the maize genome are dispersed throughout chromosomes including in gene-rich regions of chromosome arms [4–6].

TEs are a potential hazard for genome integrity as their unchecked movement will result in increasing genome size, potentially deleterious mutations and chromosome instability. Transcriptional regulatory mechanisms, including epigenetic silencing driven by DNA methylation, play important roles in the silencing of TEs [7–11]. In plant genomes, TEs are highly methylated, particularly in CG and CHG (where H is any base except G) contexts, while genic sequences tend to have lower levels of DNA methylation–especially for CHG [12–14]. As genome size increases, largely due to accumulation of LTR TEs, the overall level of CG and CHG methylation increase [13]. DNA methylation in plant genomes can be found in different contexts and is the result of different pathways [15,16]. CG methylation is largely the result of MET1 (or orthologs) and can be propagated following DNA replication due to the presence of hemi-methylated sites. CHG methylation is largely attributable to CMT3 (or orthologs) and is targeted through a self-reinforcing loop with H3K9me2. CHH methylation occurs at asymmetric sites and is often the result of RNA-directed DNA methylation (RdDM) activities. The maize genome contains high levels of CG and CHG methylation [12,17,18]. CHH methylation levels are relatively low and are often found at the edges of TEs located near genes [18,19].

The chromatin landscape can affect TEs insertion site preference through a variety of mechanisms [20]. However, the lack of active transposition for the majority of TEs in the maize genome has made it difficult to investigate the insertion site preference for most families. There are two well characterized active TIR families, *Mu* and *Ac/Ds*, which tend to land in regions of accessible chromatin with low methylation levels [21]. In contrast, many maize LTR elements are found inserted within other TEs, which could reflect a bias towards insertion into silenced chromatin [22]. Family level knowledge of chromatin influences on insertion site has been limited by the lack of consistent annotations and genome-wide chromatin data sets. In addition, the current set of TE insertion sites in maize inbred lines is a result of both the insertion site preferences of TE families and the insertions that were tolerated during selection of improved varieties.

After TEs are inserted, they are likely targeted by DNA methylation and it is possible that this silencing may spread beyond the borders of the TE, resulting in changes to flanking chromatin. There is evidence that the high levels of DNA methylation targeted towards TEs can result in increased DNA methylation at flanking regions in several plant species [23–26], potentially resulting in epialleles. These epialleles represent differences in DNA methylation levels at a genetically similar sequence in the two lines that is actually the result of a genetic change (TE insertion) nearby. These changes may represent obligatory or facilitated epialleles which require a genetic change to trigger or enable the chromatin change [27,28]. There are unresolved questions about how common the spreading of DNA methylation from TEs is and whether certain families are more likely to trigger changes in nearby regions. The level of DNA methylation flanking maize LTR families is quite variable [24]. In rice, the extent of DNA methylation flanking LTR elements may be influenced by the location in the genome, age and recombination rates [26]. There is evidence that some *Arabidopsis* TEs can trigger changes in nearby chromatin [23,25], but the relatively low number of elements and lack of high copy families have limited the ability to study variation at the family level that exists for post-insertional impacts using *Arabidopsis thaliana* as a model system.

The maize genome provides ample opportunities to study the interplay between TE families and DNA methylation. We assessed whether the >500 TE families with over 20 non-nested members in the B73v4 reference genome assembly exhibit variable profiles of DNA methylation in flanking regions. Three clusters of TE families were identified based on high, moderate or low levels of CG/CHG methylation in flanking regions. These patterns were found to be highly stable in other tissues and genotypes. The differences in DNA methylation in flanking regions were associated with different profiles of chromatin accessibility and modifications within or flanking the TEs. Polymorphic TE insertion sites defined by comparison of TE content across four maize genome assemblies [29] were utilized to monitor the likely chromatin state prior to insertion as well as the changes in chromatin that are associated with the presence of the TE. Haplotypes that lack the TE compared to those with the TE suggests that many TE families have frequent insertions within highly methylated regions, especially for LTR elements. However, a subset of TE families have a high proportion of unmethylated insertion sites. These TEs that insert within unmethylated regions frequently result in changes to DNA methylation for the flanking sequences potentially resulting in epialleles.

## Results

To evaluate the interactions between TEs and chromatin we collected datasets to document genomic variation in TE content and chromatin patterns for multiple maize genotypes (B73v4, PH207, W22, and Mo17). There are 225,000–315,000 annotated TEs in each genome that are grouped into >23,000 families (S1 Table) ([3]; [29]). DNA methylation within and near these TEs was assessed using existing or new whole genome bisulfite sequencing (WGBS) datasets (S2 Table). For each WGBS dataset the coverage and percent methylation for CG, CHG and CHH contexts was determined for 100 base-pair (bp) windows based on reads that map uniquely to the corresponding reference genome (Methods). Overall, 81% of the 2.1Gb maize B73v4 genome has at least 2X coverage but the proportion of windows annotated as TEs have slightly lower coverage (S1 Fig), likely due to the challenges of mapping to repetitive regions. The distribution of DNA methylation levels for 100bp tiles revealed bimodal distributions, especially for CG and CHG methylation, in all four genotypes (S2A Fig). Each tile was classified as unmethylated (<20%), methylated (>40%), or intermediate (20–40%) for CG and CHG methylation in each sample. The proportion of methylated tiles varies in different genomic regions (S2B Fig). For example, the proportion of unmethylated CHG tiles varies from 1.7% in TE regions to 88.3% in exons (S2B Fig). While intergenic regions (non-TE sequences located between genes) contain some unmethylated tiles, a majority are highly methylated, similar to the profile for TEs (S2B Fig). We sought to determine whether TEs might play a role in the high level of DNA methylation observed within intergenic regions.

### Different TE families exhibit distinct patterns of CG and CHG methylation in flanking regions

**Fig 1:**
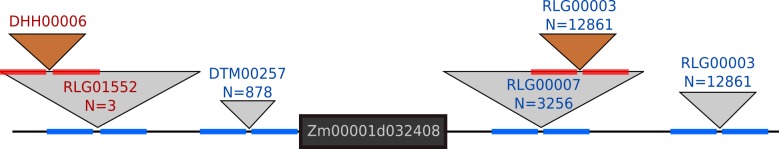
A region on chromosome 1 of the B73v4 genome from 225,749,884bp to 225,830,165bp is displayed as a schematic of genes (rectangles) and TEs (triangles). All TEs are labeled with their TE family name and family size with red text indicating small families (< 20 members) and blue text indicating large families (> = 20 members). Nested (within another TE) and non-nested elements are shown in orange and grey respectively. Flanking regions are identified by color based on whether they are outside of other TEs (blue) or located within other TEs (red). Flanking regions within other TEs are excluded from our analyses.

**Fig 2:**
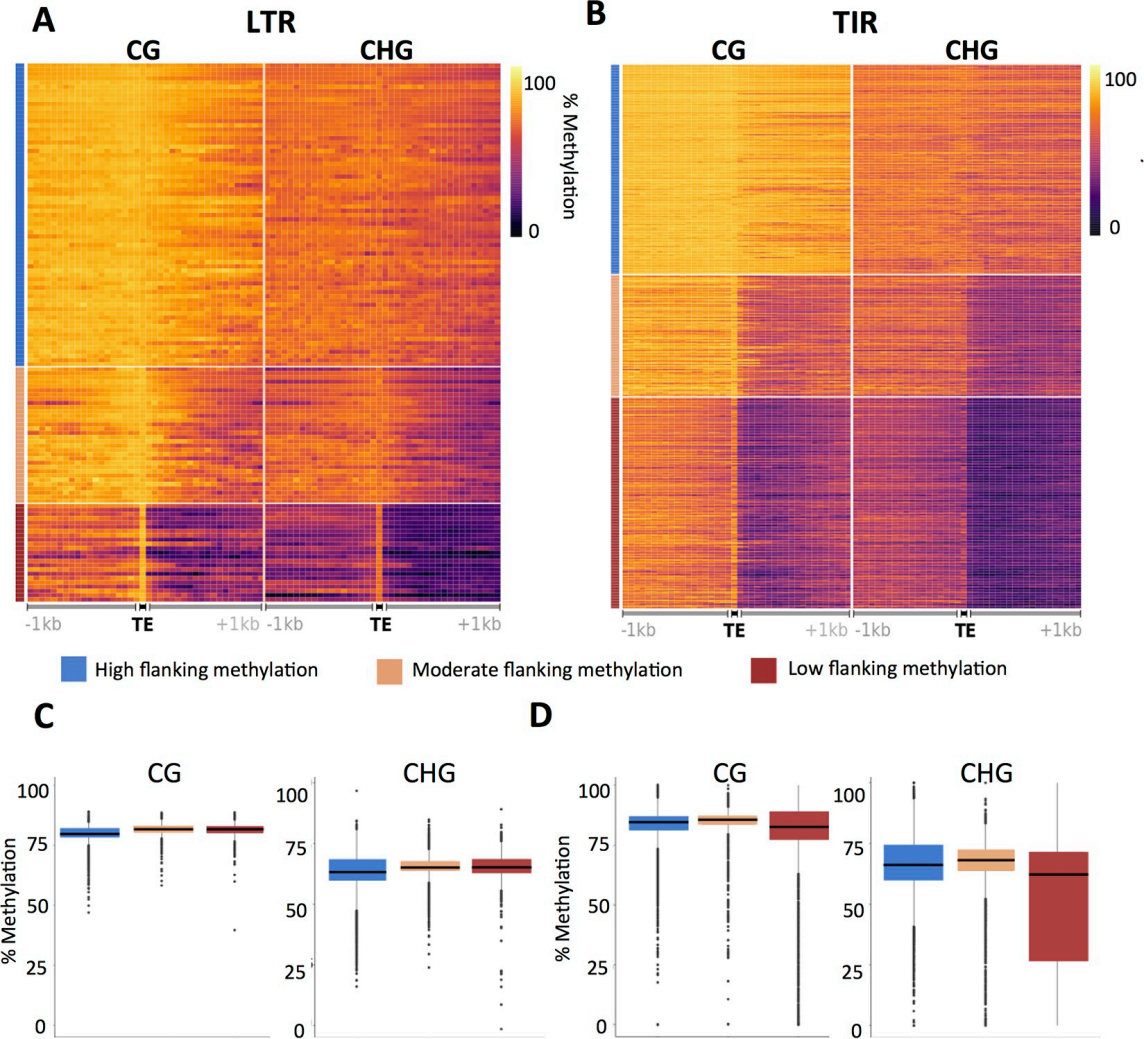
Variation in the profiles of CG and CHG methylation flanking TE families. (A-B) For 126 LTR families (A) and 438 TIR families (B) that include > = 20 non-nested annotated members the metaprofile of CG and CHG methylation was determined. Each element was oriented such that the 1kb flanking the TE with higher average methylation is plotted on the left. The profiles of CG and CHG methylation were used to perform k-means (n = 3) clustering and the profiles were plotted with a heat map. Three clusters were defined using k-means clusters of CG and CHG methylation profiles for each element and these clusters are classified as having high (H, blue), moderate (M, orange) and low (L, red) flanking CG and CHG methylation are indicated. (C-D) The average level of CG and CHG methylation within each LTR (C) and TIR (D) assigned to the clusters was determined and used to generate boxplots to investigate differences in methylation levels for the three clusters.

Previous work has classified varying patterns of DNA methylation flanking different families of LTR retrotransposons in the maize B73v2 genome [24]. This work was restricted to LTR elements and was based on a repeat-masked annotation of TEs. The availability of improved genome assemblies and annotations of intact TEs provided new opportunities to study the interplay between TE families and DNA methylation [3]. To document the variation in the profiles of DNA methylation flanking TEs present in the B73 genome we focused our analyses on non-TE genomic regions that flank transposons and initially used DNA methylation profiles for B73 shoot tissue. Each 100bp tile was associated with the nearest TE such that regions between two nearby TEs are only assigned to the closest TE. This approach excluded the flanking regions of TEs that are inserted within other elements (nested) from our analysis (Fig 1). Given our interest in comparing the profiles of different families of TEs we focused on the subset of >500 TE families with at least 20 non-nested elements for which a robust family-wide estimate can be generated (S1 Table). This resulted in profiles of DNA methylation flanking the elements for 438 TIR families and 126 LTR families in the B73v4 reference genome. Since the orientation for many TEs, especially TIRs, is not easily determined we oriented each element based on the average level of methylation in the 5’ and 3’ regions such that the side with higher CG methylation, within the 1kb flanking the TE, is always aligned on the left within metaplots. Metaprofiles of CG and CHG methylation for TIR and LTR elements exhibit different patterns, especially for regions flanking the elements (S3 Fig). Overall, there are high levels of CG and CHG methylation within the TIR and LTR elements with reduced methylation in flanking regions. The methylation decrease in regions flanking TEs is relatively gradual for LTRs while TIRs show a more distinct drop in methylation levels near the boundaries of the element (S3 Fig). CHH methylation levels are consistently low in the 1kb flanking regions for both TIRs and LTRs and therefore CHH methylation was not utilized to assess DNA methylation variation for flanking regions of TEs (S3 Fig). To assess the variability of DNA methylation patterns flanking elements in different TE families, metaprofiles of DNA methylation were generated for elements in each family with >20 non-nested members and used to perform k-means (k = 3) clustering (Fig 2A and 2B). Visualization of the profiles for the three clusters for LTR and TIR families revealed variable patterns for CG and CHG methylation in flanking regions (Fig 2A and 2B). The majority of LTR families have quite high levels of CG/CHG methylation in flanking regions with a small subset showing moderate or low flanking methylation (Fig 2A). In contrast, TIRs have many more families with lower levels of DNA methylation in flanking regions (Fig 2B). The LTR and TIR families were classified into three categories: TE families defined by consistently high-methylation flanks (H), TE families defined by partial decay of methylation levels (which includes examples in which methylation only drops to intermediate levels or examples in which the reduced methylation does not occur until >500bp from the element) classified as moderate flanking methylation (M) and TE families defined by rapid decay of methylation for at least one of the flanking regions classified as low flanking methylation (L). Although these groups have different levels of DNA methylation for flanking sequences they all have consistently high CG and CHG methylation levels over the TE body (Fig 2C and 2D).

### Consistency of DNA methylation profiles surrounding TEs in multiple tissues and genotypes

**Fig 3:**
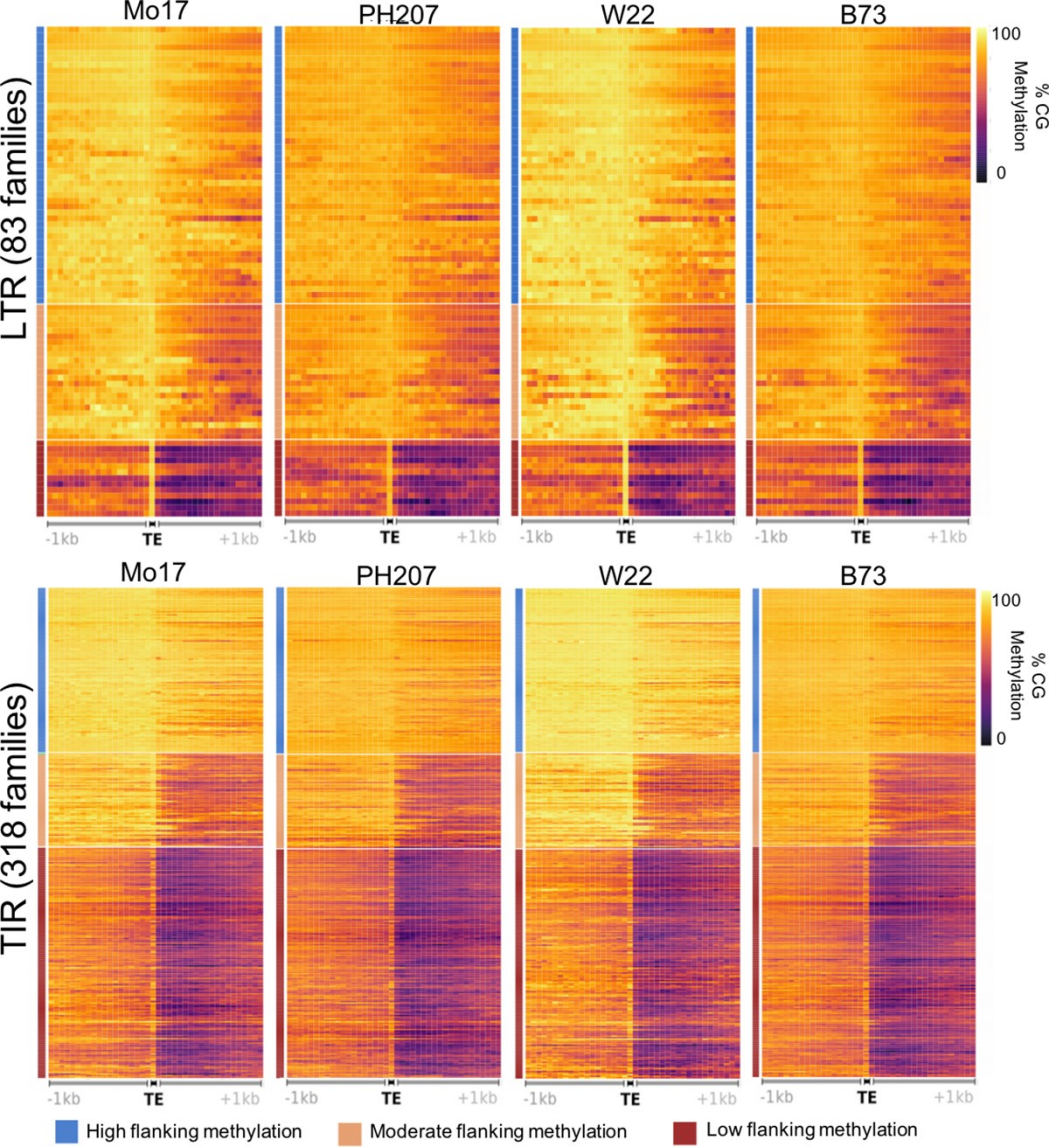
Consistency of methylation profiles surrounding TEs in different maize inbreds. For 83 LTR families and 318 TIR families that have at least 20 non-nested members in all four genotypes the metaprofile of CG DNA methylation was determined. The order of families was kept the same as in Fig 2, although a subset of the families were omitted as they did not have 20 members in at least one other genotype. The DNA methylation levels were determined based on the alignment of WGBS to the genome assembly from which it was derived and using the elements annotated within that genome. Very similar consistent patterns are also observed using CHG methylation profiles.

These metaprofiles and classifications of TE families were entirely based upon DNA methylation data for shoot tissue of B73 seedlings. The profiles of DNA methylation are very similar in other B73 tissue types, suggesting that these patterns are stable during vegetative development (S4 Fig). We also assessed the similarity of the DNA methylation patterns for TE families in the four maize genomes. There is substantial TE presence/absence variation among these four genomes [29] which results in different sizes and genomic distributions of TE families among genotypes. We generated heatmaps of DNA methylation profiles for 83 LTR and 318 TIR families with at least 20 non-nested members in all four genotypes (Fig 3). This revealed that TE families show profiles consistent with the B73 classification in other genomes (Fig 3), suggesting that the variability in DNA methylation profiles for different TE families is a property of the TE families themselves and not solely due to the collection of genomic locations for each family within B73.

### Variable flanking methylation levels are associated with additional chromatin changes within or flanking TE families

**Fig 4:**
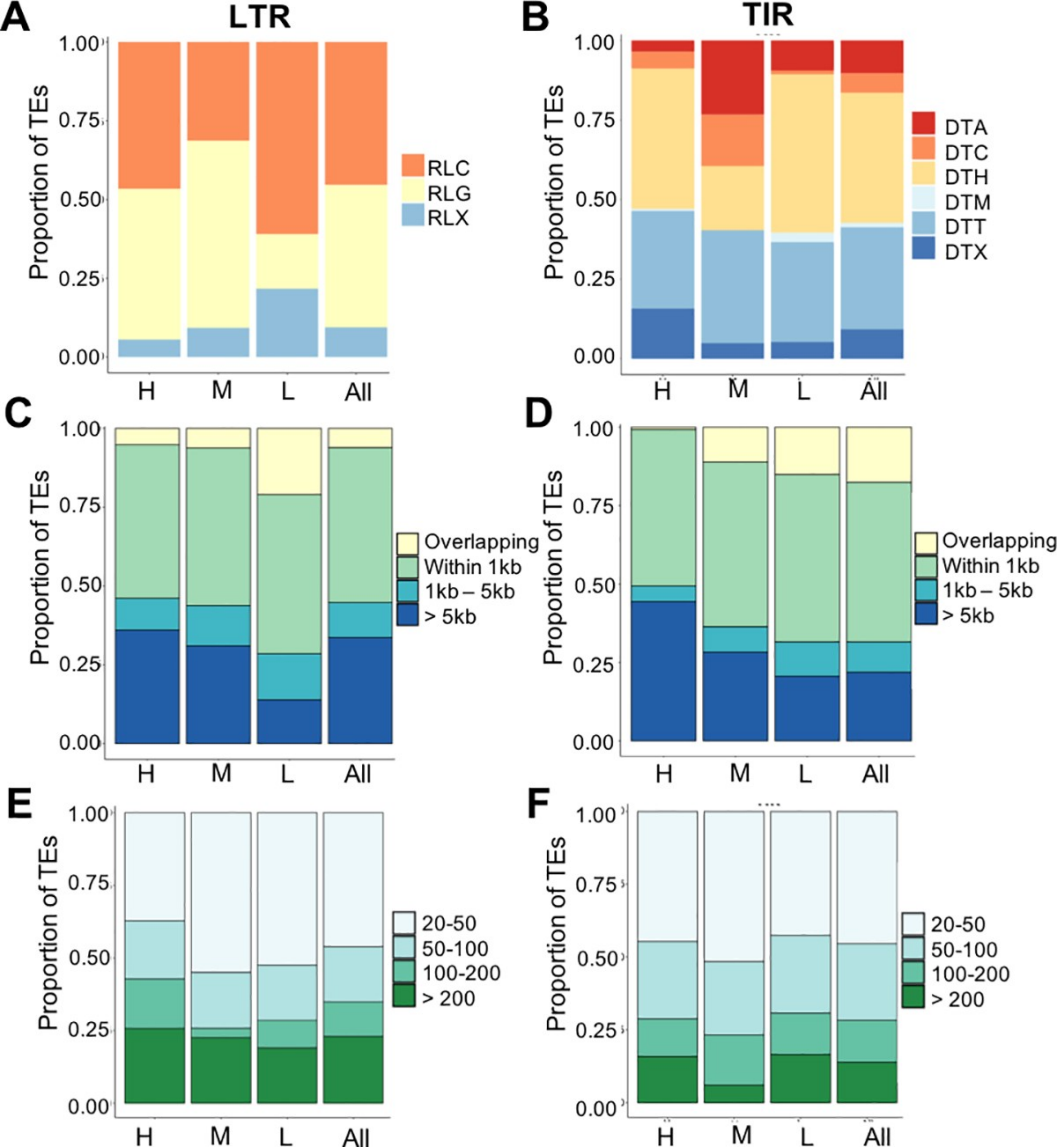
Analysis of attributes for members of TE families of high, moderate and low flanking CG/CHG methylation. (A-B). The proportion of superfamily designations for TEs classified as having high, moderate or low flanking methylation were compared to the proportion for all TE families. LTR elements (A) are classified into copia (RLC), gypsy (RLG) or unclassified (RLX) superfamilies. TIR elements (B) are classified as hAT (DTA), CACTA (DTC), PifHarbinger (DTH), Mutator (DTM), Tc1/Mariner (DTT) or unclassified (DTX). (C-D) For all elements of TE families classified as having high, moderate or low flanking methylation the distance to the nearest gene was determined and binned overlapping, within 1kb, 1-5kb or >5kb. The proportion of elements with varying proximity to genes was compared for the different clusters of TEs relative to all LTR or TIR elements. (E-F) A similar analysis was done to compare the proportion of families with different copy numbers in each cluster to all LTR (E) or all TIR (F) elements. Family size was classified into 4 groups: 20–50 members, 50–100 members, 100–200 members, and >200 members from light to dark color respectively.

The observation that TE families exhibit distinct patterns of CG and CHG methylation in flanking regions led us to investigate several features of the families that might be associated with this variation. Each LTR and TIR family is associated with a specific superfamily. LTRs with low flanking methylation are depleted for RLG (gypsy) families and enriched for RLC (copia) and RLX (unknown) families relative to the other groups (Fig 4A). The TIR families with moderate flanking methylation are enriched for DTC (CACTA) and DTA (hAT) families (Fig 4B). The proximity to genes for the TEs in the three groups suggests that the TEs with high levels of flanking methylation are slightly enriched in TIRs located far from genes, but there are also many TEs within this group that are near genes (Fig 4C and 4D). The number of elements per family was assessed to determine if there was any enrichment for large families in high, moderate, and low flanking methylation patterns. There were no striking trends in terms of the size of TE families in the three groups, with high, moderate, and low flanking methylation groups showing a blend of family copy number (Fig 4E and 4F). While there are some differences in the properties of families in the groups there are no defining factors that can be used to predict the behavior of DNA methylation in the regions flanking LTR or TIR families.

**Fig 5:**
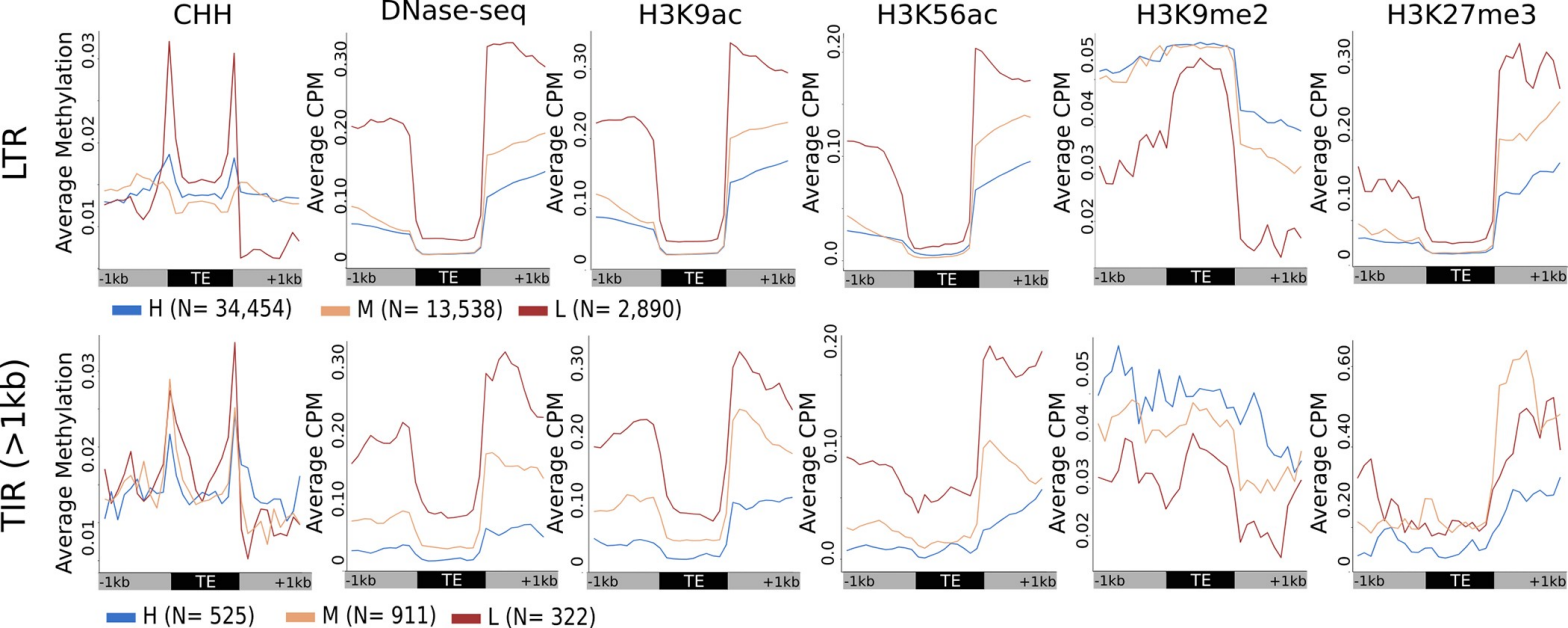
Analysis of chromatin within and surrounding the three clusters of TEs defined based on flanking levels of CG and CHG methylation. The relative abundance of CHH methylation, chromatin accessibility (DNase-seq and histone modifications H3K9ac, H3K56ac, H3K9me2 and H3K27me3 were determined within and flanking TEs that were classified as having high, moderate or low flanking methylation patterns. The CHH methylation was determined for 100bp tiles and the average level within the H, M and L categories was calculated. For the remaining chromatin marks (ChIP and DNase), the average CPM for each 100bp bin within the categories H, M and L was calculated. The top set of plots show the metaprofiles for LTR elements (N = 50,882) in the three clusters with high (blue), moderate (orange) and low (red) indicated with different colors. The lower set of plots show the metaprofiles for the three clusters of TIR elements (N = 1,758) only using elements >1kb in length. Number of elements in each group indicated next to key.

The high, moderate, and low flanking methylation classifications were defined based upon patterns of CG and CHG methylation in flanking regions. We assessed how these groups varied for other chromatin modifications within and flanking these families (Fig 5). As TIR families often include many quite small non-autonomous elements and the resolution of some chromatin data can be limited, we focused on the subset of TIR elements with a length greater than 1kb (Fig 5). In contrast, the vast majority (99.97%) of LTR elements are over 1 kb and therefore we included all LTR elements. There are interesting dynamics for CHH methylation at the edges of TEs classified into the different groups. LTR families with low methylation in flanking regions show a striking peak of CHH methylation at the edges of the TE while the other classes of LTR elements have lower levels of CHH. All three groups of TIR elements exhibit an increase in CHH methylation at the edges for TE families in all three groups with the strongest enrichment in the families with low levels of methylation in flanking regions. This suggests that RNA-directed DNA methylation is most active at the edges of TEs that are located near unmethylated DNA as previously noted in maize [19]. The evaluation of chromatin accessibility (DNase-seq) [30] and histone modifications [12,30,31] show differences among the TEs classified as having high, moderate or low flanking methylation. Due to the high methylation over TE bodies, we anticipated low levels of accessibility within TE bodies. As expected, chromatin accessibility is quite low within the element itself for all types of LTR and TIR elements, with somewhat elevated levels for TIRs with low levels of flanking methylation. There are more pronounced differences in chromatin accessibility for the regions flanking the elements of families with low levels of CG/CHG methylation (Fig 5). H3K9ac and H3K56ac tend to be associated with active chromatin and would therefore be expected to have an inverse relationship with methylation trends. These histone acetylation modifications tended to be quite low within all LTR elements but showed variable levels in flanking regions (Fig 5). For TIRs, there are differences for these histone acetylation modifications within, and in flanking regions, in the three groups (Fig 5). H3K9me2 is typically associated with highly methylated silenced chromatin and is enriched within and flanking LTR elements that are classified as having high or moderate flanking CG/CHG methylation. There is less evidence for strong enrichment of H3K9me2 within TIR elements relative to flanking sequences and there seems to be a depletion of H3K9me2 in the region immediately flanking the TIRs of elements with low levels of flanking CG/CHG methylation (Fig 5). H3K27me3 is often associated with developmental silencing of gene expression and we see relatively low levels of this modification within TEs. There are higher levels of H3K27me3 in the regions flanking LTR elements that are classified as having low levels of flanking CG/CHG methylation and lower levels of enrichment for H3K27me3 in regions flanking TIR elements with moderate or low methylation for the flanking regions. Together, these observations suggest that different subsets of TE families have distinct profiles of chromatin and DNA methylation within and near the elements.

### TE Expression is not strongly related to clusters defined by DNA methylation

The expression level of TE families in the different clusters was assessed to determine whether variable expression patterns could be associated with the CG/CHG methylation trends observed flanking different TE families. Many TE families exhibit detectable levels of expression in RNAseq datasets [32]. An approach to document the per-family expression of TEs that utilizes unique and multi-mapping reads [32] was used to determine the expression level of all TE families in a panel of 23 tissues [33]. We compared the proportion of TE families with detectable expression (average RPM > 1) for each of the clusters (S5A and S5B Fig). Among the TE families with sufficient copy number to be classified based on flanking DNA methylation levels, we see a slightly higher rate of expression for the TIR families with low flanking methylation (S5A and S5B Fig). However, there are also a number of TE families with high methylation for flanking regions that show expression as well (S5 Fig). For the TE families that exhibit detectable expression (115 LTR and 239 TIR families) we assessed the tissue-specific patterns of expression (S5C and S5D Fig). Some families in each group of high, moderate, and low flanking methylation show expression across many tissues while most families exhibit more dynamic patterns. However, there was not a clear association between clusters of elements defined by flanking DNA methylation and TE expression across tissues.

### TE family level variability for DNA methylation levels at insertion sites

In the previous sections we focused on classifying TE families with >20 members inserted into low-copy regions based on flanking DNA methylation patterns. This revealed differences between TIR and LTR families and revealed clusters of TEs that exhibit differences in chromatin and TE expression patterns. This variation may be the result of differences in preference for DNA methylation level at the insertion site for TE families or due to differences in how TEs influence DNA methylation of nearby regions once they are inserted. A comparative analysis of structural annotations of TEs in the assembled genomes for four maize inbred lines including B73, PH207, W22, and Mo17 resulted in the documentation of shared and non-shared TE insertions [29]. The characterization of TE polymorphisms among these four genotypes [29] allowed us to evaluate potential DNA methylation insertion site preferences for TE families as well as the changes in DNA methylation that accompany the presence of the TE. In this analysis we are assuming that the DNA methylation state for the haplotype lacking the TE reflects the DNA methylation state prior to insertion which is likely true in most instances. Indeed, the analysis of examples for which there is a TE insertion in one haplotype but empty sites in the other 3 haplotypes reveals that over 93% of these sites are consistently methylated or unmethylated for all three empty sites.

**Fig 6:**
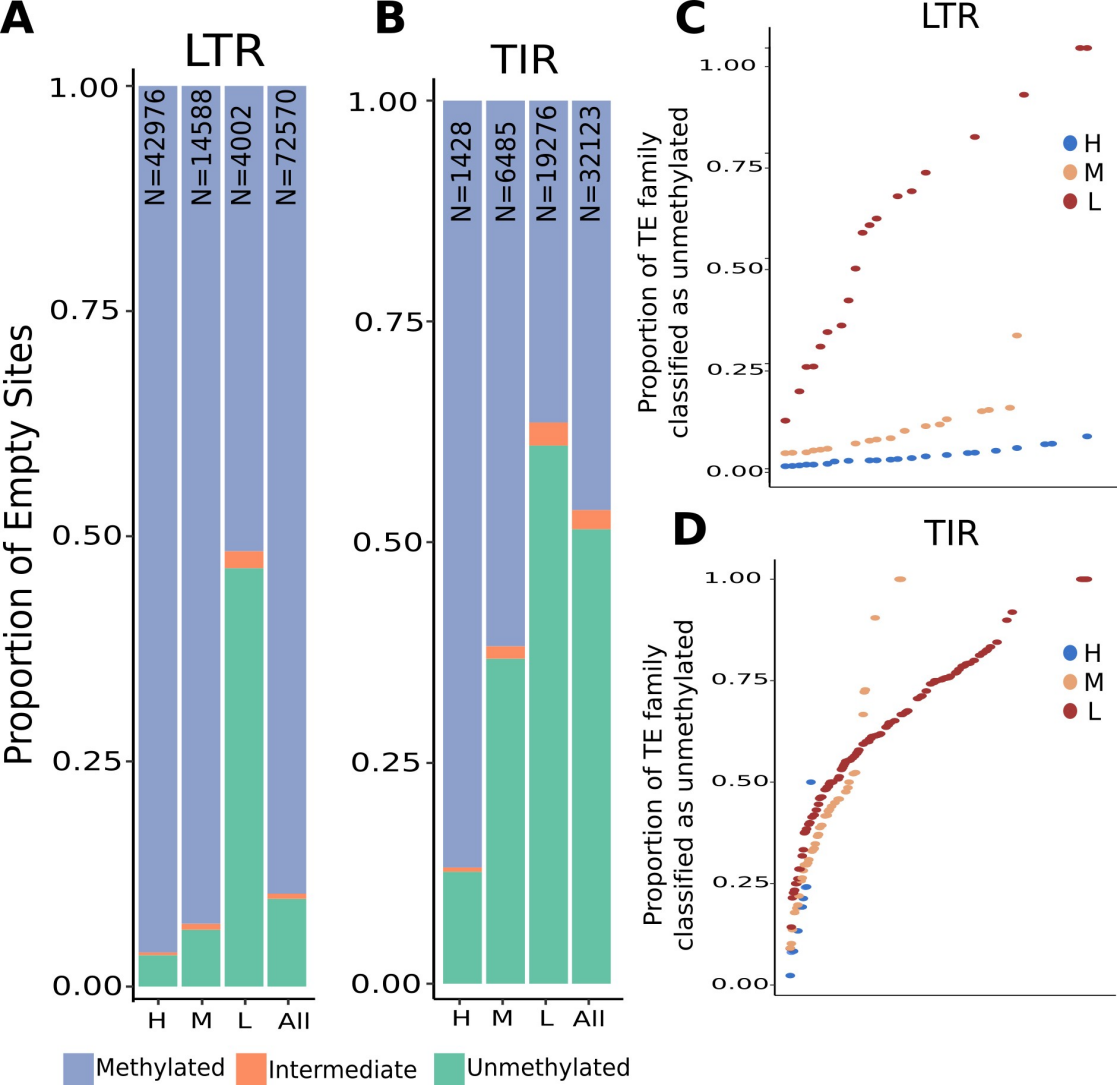
Levels of CG DNA methylation at empty site haplotypes. There are 75,570 LTR (A) and 32,123 TIR (B) TE polymorphisms for which there is WGBS data for the haplotype that lacks the insertion (empty site). The empty sites were classified based on CG methylation level as unmethylated (<20%), intermediate (20–40%), or methylated (>40%) and the distribution of these three groups is shown for all TEs as well as TEs in families classified as having H, M or L flanking methylation. (C-D) For each TE family with at least 10 empty sites the proportion of CG unmethylated empty sites was determined and used to rank order the families.

There are 69,292 polymorphic TE insertions among the four genotypes that have highly conserved sequence in the 200bp flanking the TE and provide the opportunity to compare DNA methylation levels in these regions without confounding flanking sequence level variation. The haplotype without the TE was defined as the “empty site” as there is no TE insertion in this haplotype but at least one other haplotype has an insertion at this site (S6 Fig). The DNA methylation state for the 100bp tile containing the empty site was determined for 36,285 LTR insertions and for 16,061 TIR insertions (Fig 6A and 6B). LTRs tend to exhibit a strong enrichment for high methylation at the insertion site (89.7% of empty sites are methylated) while the empty sites of TIR insertions are less often methylated (46.4% of empty sites) (Fig 6A and 6B). There are differences in the proportion of unmethylated empty sites for the TEs classified into high, moderate, and low flanking methylation patterns. TEs from families with low flanking methylation have a higher proportion of unmethylated empty sites while TEs from families with high flanking methylation are more frequently methylated at empty sites. This suggests that chromatin insertion site preferences may explain a large portion of the flanking methylation profiles for TE families. For all TE families with at least ten empty sites the proportion of unmethylated empty sites was assessed for each family (Fig 6C and 6D). All of the LTR families with high levels of flanking methylation have >90% of the insertions located within DNA that is already methylated. In contrast, LTR families with lower levels of flanking methylation exhibit variable levels of methylation at insertion sites (Fig 6C). The TIR families exhibit more variation for the proportion of insertion sites that are methylated (Fig 6D). Since TIR DNA transposons can be mobilized through cut-and-paste transposition it is likely that a subset of the TIR empty sites may reflect excision of elements rather than the haplotype prior to insertion. It can be difficult to identify true excision sites but in many cases an excision results in elimination of the target site duplication sequence we can identify a subset of TE polymorphisms that are likely enriched for excision events. In cases in which a TE was inserted and then excised, it could influence DNA methylation of the haplotype through epigenetic memory of the chromatin marks. We assessed whether there are differences in the frequency of methylated or unmethylated empty sites for the excision sites relative to new insertions. There are not major differences in the frequency of unmethylated empty sites for the excision events compared to novel insertions (S7 Fig).

### TEs can result in changes to methylation in surrounding regions

**Fig 7:**
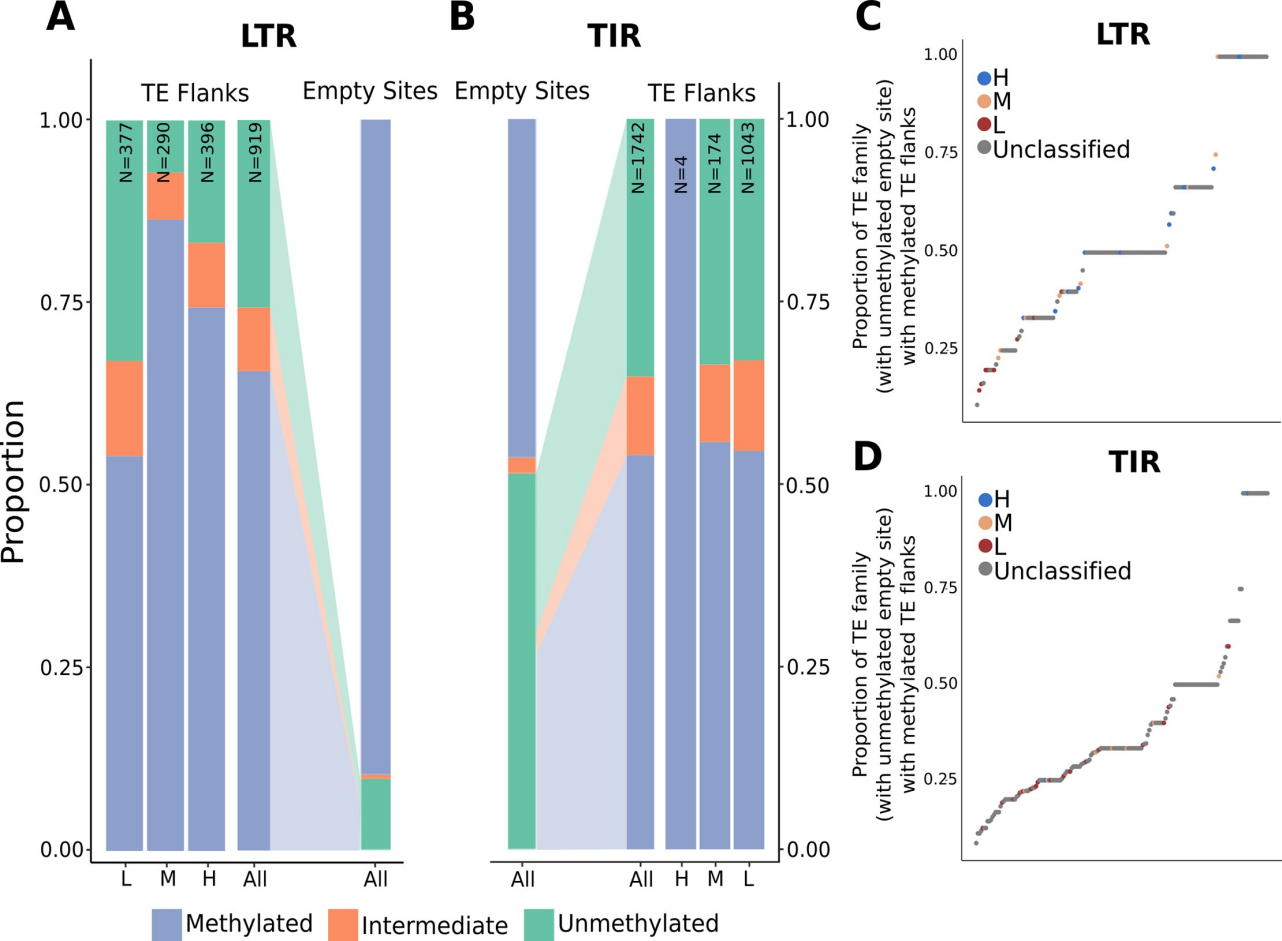
Analysis of CG DNA methylation changes induced by TEs. (A-B) The subset of TEs that are located within CG unmethylated empty sites could assessed for changes in levels of CG methylation in flanking regions. There are 919 LTR elements (A) and 1742 TIR elements (B) that represent insertions into unmethylated empty sites for which there is CG methylation data for the regions flanking the TE. The proportion of these TEs that show methylation, based on the average CG methylation of the TE flanks, was determined for all of these sites as well as the subsets that are near TEs belonging to families classified as having H, M or L flanking CG and CHG methylation. For the set of LTR (C) or TIR (D) families that have at least four insertions into CG unmethylated regions, the proportion of family members that gain CG DNA methylation was determined and used to rank order the families. Each family was color coded based on it’s classification for H, M or L flanking methylation. The unclassified families did not have enough elements to be classified as H, M, or L.

In addition to assessing methylation at insertion sites, we were also interested in documenting what happens to the chromatin at unmethylated empty sites after the TE inserts. In the haplotype with the TE insertion it is possible that the regions flanking the TE would remain unmethylated. Alternatively, the presence of the TE could be associated with an increase of DNA methylation in these flanking regions. This would result in variable methylation for conserved regions between two inbreds that are the result of the nearby genetic change (i.e. TE insertion). The level of DNA methylation flanking the TE (the 100bp tiles on either side of the tile containing the polymorphic TE as in S6 Fig) was assessed for TEs with unmethylated empty sites (Fig 7A and 7B) using the TE polymorphism and DNA methylation data for all four genotypes. For the majority of the loci (54.3% of TIR insertions and 65.6% of LTR insertions) that could be assessed, there is evidence for an increase in DNA methylation in at least one flank associated with the TE insertion into unmethylated empty sites. As expected, TEs belonging to families with low flanking methylation were less likely to be associated with gains of methylation in flanking regions relative to those with high and moderate flanking methylation. The analysis of multiple members of the same TE family revealed that 12.5% of families exhibit gains of methylation for all members of the family while there are other families with low or moderate frequencies of elements that trigger methylation gains (Fig 7C and 7D).

The observation that some unmethylated sites gain DNA methylation following the insertion of the TE while others do not could reflect different properties of specific insertions or could suggest a stochastic nature for methylation spreading at the edges of transposons. We looked at the patterns of methylation at 88 unmethylated empty sites in B73 with TEs present in all three of the other genomes to assess whether the patterns were consistent among genotypes. The majority (86%) of these sites gain methylation in flanking regions for at least one genotype. At most of these loci (83.3% for TIRs and 78.5% for LTRs) DNA methylation is gained in multiple genotypes, often in all three (S8 Fig). An example locus (S8 Fig) illustrates the similar gain of DNA methylation for the haplotypes containing DNA methylation. This suggests that the subset of TEs for which methylation is gained in flanking regions represent effects that are consistent across genotypes rather than reflecting stochastic variation triggered by the TE.

**Fig 8:**
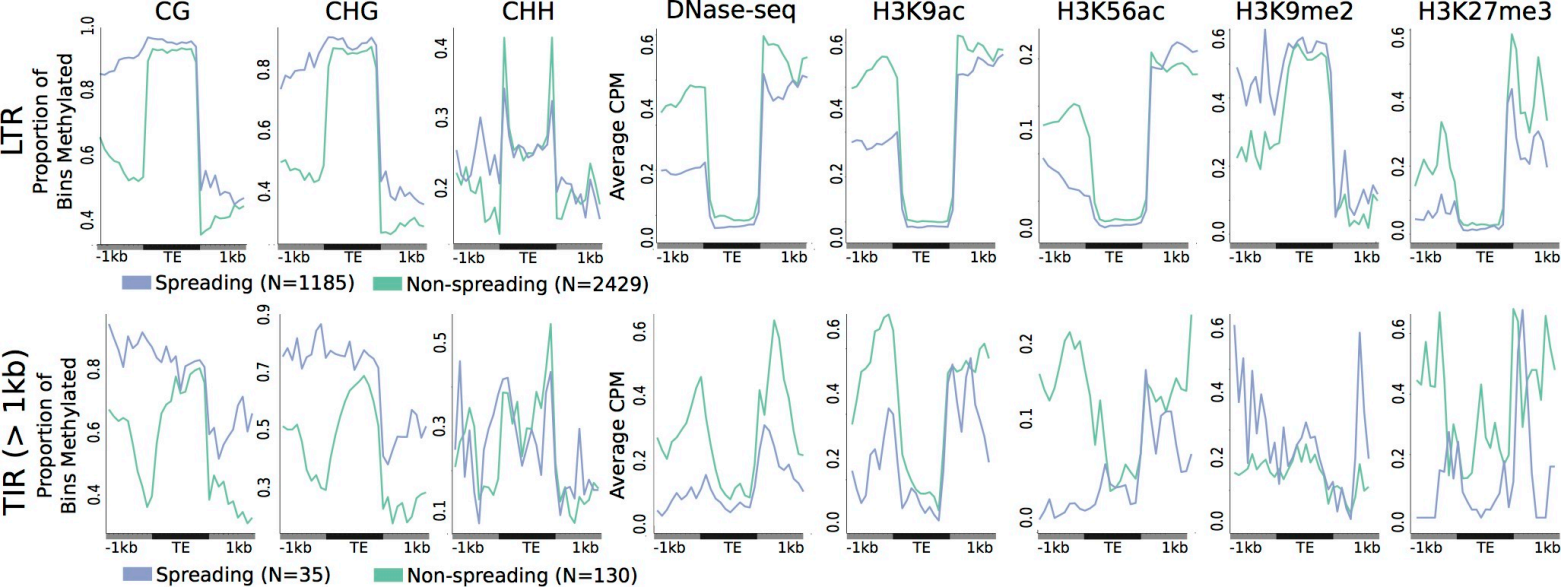
Chromatin profiles at elements with or without spreading of DNA methylation. For LTR or TIR elements that are inserted into CG unmethylated empty sites we assessed the chromatin profiles based on the proportion of bins methylated (>40% for CG/CHG and >2% for CHH) or average CPM (ChIP-seq and DNase-seq) for elements with spreading (purple) or without spreading (green) of DNA methylation in B73. Elements are oriented with the highest average level of methylation on the left. Number of elements in each group is indicated next to the key.

We investigated whether there are differences in the chromatin profiles for TEs that exhibit changes in methylation for flanking regions compared to those without changes (Fig 8). As there is only chromatin modification data available for B73 this analysis focused on the 4,791 TIR and 3,649 LTR elements that are present in B73 but have unmethylated empty sites in other haplotypes. There are not large differences in the level of CG or CHG DNA methylation within the elements that exhibit spreading compared to those that do not (Fig 8). A visualization of DNA methylation near elements classified as spreading or non-spreading (Fig 8) suggests that changes in the average level of DNA methylation are more prevalent on one side of the element relative to the other. For elements with both flanking regions classified as unmethylated that show evidence of spreading the spreading is only observed on one flank for 54% while the remaining 46% of sites exhibit spreading for both regions. For the LTR elements we were able to assess the profiles of DNA methylation when we use the genomic orientation of the element (rather than orienting based on which side has higher DNA methylation (S9 Fig)). This reveals relatively similar differences in methylation for the 5’ and 3’ flanks for LTR elements. Together with the results in Fig 8 this suggests that spreading often occurs on one side of the element but that this is not defined by the orientation of the elements, at least for LTRs. The non-spreading LTR elements exhibit a stronger enrichment for CHH methylation at the edges of the elements relative to spreading LTR elements and is quite low in the regions flanking these non-spreading elements (Fig 8). The TEs without evidence for spreading of DNA methylation into flanking regions tends to have higher levels of chromatin accessibility, H3K56ac, H3K9ac and H3K27me3 in flanking regions but very little difference within the elements themselves (Fig 8). Overall, we do not see strong evidence for differences for the chromatin within the body of TEs that exhibit spreading of DNA methylation compared to those that do not but there are differences in some chromatin modifications in the regions flanking these two sets of TEs. We also investigated the attributes of TEs with, or without spreading, of DNA methylation and did not find major differences in the distance to genes, superfamily designation, family size, length, or age (S10 Fig). We also do not see any differences in the frequency of CG, CHG or CHH sites within the flanking regions or TEs bodies for the elements classified as spreading or non-spreading (S11 Fig).

## Discussion

The maize genome provides opportunities to study variation in how different TE families interact with DNA methylation. In particular, the presence of abundant TEs, including many in moderate to large families, enables family level analyses of the profiles of DNA methylation and chromatin. In addition, frequent TE polymorphisms among inbred lines provides insights into the variability for both chromatin influences on insertion site preference and potential spreading of methylation following insertion. We observe distinct profiles for DNA methylation and chromatin surrounding TE families, highlighting the importance of not averaging profiles for all TEs together. It is likely that different TE families have adopted distinct strategies that enable their survival and proliferation within eukaryotic genomes which will result in distinct behaviors relative to chromatin and epigenetic regulatory mechanisms.

The TEs of the maize genome are highly methylated [17,34,35]. This methylation likely originates from targeted *de novo* DNA methylation to transposons followed by efficient maintenance of CG and CHG methylation [36–39]. Metaplots of DNA methylation show very high levels of CG and CHG methylation within LTR and TIR elements [17]. However, the level of DNA methylation at the edges of elements remains somewhat elevated and does not decay for several hundred base pairs. There are several factors that could influence the levels of DNA methylation near TEs. First, DNA methylation and associated chromatin modifications could influence the insertion sites for TEs. Second, TE insertions could disrupt existing chromatin and recruit DNA methylation that would spread to flanking regions. Third, the act of selection upon elements, and the chromatin changes they cause, could influence the patterns in the extant elements. We investigated each of these forces as we considered how TEs shape the methylome of maize.

### TE insertion site preferences for chromatin state

The transposase or integrase enzymes of TIR and LTR transposases often include domains that can interact with histone modifications [20]. This provides the opportunity for TEs to insert within largely silenced, or active, regions of the genome. There are likely trade-offs for the TE to these strategies. Insertion within active regions likely increases the potential for TE expression and subsequent transposition. However, it can also result in higher mutation load and could allow for more efficient recognition and silencing by the host genome. In contrast, insertion within silenced regions is much less likely to result in deleterious mutations and could allow for TEs to attain very high copy number but may not allow for continued expression/mobility from these silenced sites [20,40]. Alternatively, TEs may insert at random sites into the genome and subsequent selection against insertions within genes could result in biased accumulation within silenced chromatin for extant elements.

To document the insertion site preferences for TE families it is most useful to have on-going transposition activity. This enables researchers to collect large numbers of new insertion sites and assess sequence or chromatin state enrichments represented by these loci. In maize, large numbers of insertion sites for *Mu* and *Ac/Ds* have been generated [41,42]. The analysis of chromatin accessibility and DNA methylation at these sites suggests that both of these TIR families have a strong preference for DNA that is accessible and unmethylated [21]. Although LTR elements comprise the majority of the maize genome, there are no families with known on-going transposition. This has limited our ability to assess the insertion site preferences for these families. There is evidence that some plant LTR elements, such as *Tos17* in rice, can exhibit a preference for active chromatin [43]. However, other LTR elements seem to have a preference for inserting into other, highly methylated elements [22]. These preferences may even change in closely related species [44].

Here we utilize site-defined TE polymorphisms between four maize inbreds to estimate chromatin preferences for different TE families. This type of analysis is potentially confounded by two factors. First, we are assuming that the DNA methylation levels are stably inherited and that the DNA methylation state at the haplotype lacking the TE represents the ancestral state. In general, regional levels of DNA methylation are quite stably inherited [45–48] and it is likely that for the majority of loci, the DNA methylation at the empty site will reflect the ancestral state. In addition, we are assuming that the haplotype without the TE represents the ancestral sequence state, which is likely true for LTR elements. However, for a subset of TIR elements this could reflect a perfect excision event. Second, the extant set of TE polymorphisms reflect insertions that have occurred and not been eliminated due to selection. Insertions within highly methylated regions may be less likely to result in deleterious alleles. Therefore, the existing set of loci with polymorphic insertions may be enriched for insertions into methylated DNA. In addition, for TIR elements a subset of the polymorphisms could be the result of excision events rather than novel insertions. Despite this, we do find substantial variation among TE families. While there are many families for which all, or the majority, of empty sites are highly methylated there are also examples of families that primarily have unmethylated empty sites. In total, there are 41 families for which >75% of empty sites are unmethylated. Importantly, many of the families that are enriched for unmethylated empty sites are classified into the low-flanking methylated group. This analysis of the DNA methylation patterns at empty sites suggests that for at least a subset of TE families, particularly within LTRs, there is a preference for insertions into highly methylated DNA which limits our ability to assess the impact of insertions upon DNA methylation spreading. While the use of natural variation data to assess the presumptive chromatin of the insertion site can be difficult to unambiguously interpret, it does provide evidence for a subset of examples where there is family specific variation for preference of insertion into methylated or unmethylated DNA.

### TE influences on nearby chromatin

A subset of TE insertions will occur in unmethylated DNA. This establishes new boundaries between methylated and unmethylated DNA. Given that the majority of unmethylated DNA within the maize genome occurs within or near genes it is likely important to regulate the extent to which the TE insertion will alter chromatin of nearby sequences. Prior studies in several plant species have identified evidence that TE insertions can result in increased DNA methylation at flanking sequences [23,25,26,38]. This has been termed “spreading” of DNA methylation although it is not clear if this truly represents a continuous spread or simply a disruption of chromatin that allows DNA methylation to occur. In rice, the extent of spreading depends on several factors including family, age of the insertion, genomic location, and TE body methylation [26]. However, we see little evidence that these variables are associated with spreading vs non-spreading elements in maize (S9 Fig). Polymorphic TEs among 140 *Arabidopsis thaliana* accessions found 50% of TEs surveyed result in DNA methylation spreading to ~300bp from the TE boundary [25,49]. Similarly, we find 54% of TIRs and 65% of LTRs that insert within unmethylated regions have high levels of flanking methylation.

We attempted to look at the distance of spreading but found this to be a complex issue. To assess spreading, or gain, of methylation it is necessary to focus on previously unmethylated regions. The vast majority of the maize genome is highly methylated with small patches of unmethylated DNA. Often when TEs insert into unmethylated DNA they are landing within a several hundred bp patch of unmethylated DNA that is flanked by methylation or in the region in between methylated DNA and an unmethylated gene. Insertions into unmethylated DNA that trigger the gain of DNA methylation often result in high levels of methylation all the way to the next methylated domain. However, the extent of DNA methylation gains in regions between the insertion site and a nearby gene is much more limited. The exact mechanisms that determine whether or not DNA methylation spreads from a TE to nearby sequence are not well characterized. Previous analyses focused on maize LTRs suggest that families with high levels of CHH methylation and 24 nucleotide small RNAs have the least spreading [19,50]. In contrast LTR families that lack CHH methylation over the LTRs are more likely to exhibit spreading. This could suggest that targeting of the RNA-directed DNA methylation machinery to TE edges could provide precise targeting and specification of boundaries. This RNA-directed CHH methylation that is found at the edge of many LTR elements may act to prevent the spread of euchromatin into TEs as well as preventing the potential spread of DNA methylation of other heterochromatin marks to flanking regions. This would be an important property that could provide a mechanism by which large, complex genomes could enable partitioning of heterochromatin and genic regions. In contrast, elements that lack RNA-directed DNA methylation may have DNA methylation maintenance and targeting mechanisms that are dependent upon histone modifications such as H3K9me2 [51,52] and these types of elements may be more likely to influence chromatin and expression of nearby genes. The proliferation of these types of elements may have more consequences for the organism. In this study, we note that there are differences for a number of histone modifications within transposable elements that have high or low levels of flanking DNA methylation.

The spreading of DNA methylation near polymorphic TE insertions can result in potential epialleles [27,53–55]. In these cases, flanking regions with highly similar sequence exhibit differences in DNA methylation. This difference is likely triggered by the TE insertion but can result in differential availability of the sequence to transcription factors or machinery. Indeed, the TEs that trigger spreading account for a subset of the differentially methylated regions identified in contrasts of maize genomes [56]. Importantly, this would also predict that TE insertions that trigger spreading of DNA methylation would also have greater potential to trigger changes in the expression of nearby genes. The dynamic and potentially variable nature of the spreading could be quite important as well. In some cases of well-characterized epialleles such as *Agouti* in mice [57], a sex-determination locus in melon [58] and *Ufo-1* in maize [59] there is evidence that a TE can lead to variable levels of spreading of chromatin that influence traits. A deeper understanding of the factors that trigger the spreading of DNA methylation and the consequences of this spread will be important as we seek to understand how TEs shape gene regulatory diversity within plant species.

## Methods

### Annotation of genes and TEs

Whole genome assemblies for B73 (Zm00001d) [60], W22 (Zm00004b) [21], Mo17 (Zm00014a) [61], and PH207 (Zm00008a) [62] were used for genome-wide analyses. All analyses were done on assemblies of chromosomes 1–10 while all scaffolds were disregarded due to the inability to assess these regions across genotypes. Filtered structural TE annotations [29] were used (available at https://github.com/SNAnderson/maizeTE_variation).

### Polymorphic TE identification [30]

Identification of shared and non-shared elements was determined through pairwise comparison between four maize inbred lines (B73, W22, PH207, and Mo17). Search windows were defined by the closest, non-overlapping genes to the query TE with a syntelog in the genome being assessed. For comparison, 400bp flanking tags were extracted for each annotated TE in the genome (for each genome assessed) centered at the start and end coordinates. These flank tags were mapped to the other genomes with use of BWA-MEM (Li and Durbin 2009) in paired-end mode. Further characterization was performed on those elements with tags mapped completely within the search window. Non-shared site-defined TEs were defined by the unique mapping of both flank tags to the window with a soft-clipped region which matches the flanking regions of the TE (does not include TE sequence). Site-defined TEs were required to maintain an absolute distance between the right and left sequence that is less than twice the TSD length of the superfamily. This resulted in a total of 69,292 non-shared site-defined elements across all pairwise comparisons used for analyses. When assessing TE polymorphisms between B73 and W22, the TSD-specific sequence was found flanking the B73 TE and the predicted W22 insertion site in 73% of cases. The analysis of identical-by-sequence genomic regions supports the high accuracy of the TE polymorphism calls [30].

### WGBS

In this study we generated novel WGBS data for B73, W22 and PH207 samples and utilized previously generated WGBS for B73 and Mo17 [SRR850328 [50]] (see S2 Table for details). For B73 and PH207 shoot seedlings (slightly prior to V1) were grown for 6 days and root tissue was separated from above ground tissue (shoot) for collection of 3 biological replicates. For WGBS DNA from the three samples was pooled and 1ug of DNA was sheared to a size of 200-300bp. These DNA fragments were then used to construct a whole-genome bisulfite sequencing library using KAPA library preparation kit (KK8232). Briefly, the DNA fragments were subjected to end repair, A-tailing, adapter ligation and dual-SPRI size selection following manufacturer’s instructions. The resulting library, which has a size between 250bp and 450bp, was treated with bisulfite sodium so that unmethylated cytosines could be converted to uracil using Zymo EZ DNA methylation lightning kit (D5031). The KAPA HiFi HotStart Uracil + (KK2801) was used in the PCR reaction with the following program: 95°C/2min, 8 cycles of 98°C/30s, 60°C/30s, 72°C/4min, and a final extension step at 72°C for 10 min. For B73 and W22 leaf tissue, plants were grown to V3 and blade tissue from the third leaf was collected for at least 2 biological replicates. DNA was pooled to generate 20ul of sheared DNA. The DNA fragments were then used to construct a whole-genome bisulfite sequencing library using the Accel-NGS Methyl-Seq DNA Library Kit (30024). Briefly, the DNA fragments were subjected to BiSulfite conversion, denaturation, adaptase, extension, and ligation following manufacturer’s instructions using the Methyl-Seq Set A Indexing Kit (36024). Finally, the PCR enriched library was cleaned up using SPRI beads. The library was sequenced using Illumina HiSeq2000 with the paired-end mode and 100 cycles. The WGBS data set has been deposited into NCBI under accession numbers SRR873827 and PRJNA527657.

Trim_glore [63] was used to trim adapter sequences and read quality was assessed with the default parameters and paired-end reads mode. Reads that passed quality control were aligned to the corresponding genome (B73v4, PH207, W22, or Mo17) using BSMAP-2.90 [64], allowing up to 5 mismatches and a quality threshold of 20 (-v 5 -q 20). Duplicate reads were detected and removed using picard-tools-1.102 (“Picard Tools–By Broad Institute” n.d.) and SAMtools [65]. Conversion rate was determined using the reads mapped to the unmethylated chloroplast genome. The resulting alignment file, merged for all samples with the same tissue and genotype, was then used to determine methylation level for each cytosine using BSMAP tools. Methylation ratio for 100bp non-overlapping sliding windows across the B73v4 genome in all three sequence contexts (CG, CHG, and CHH) was calculated (#C/(#C+#T)). Each 100bp window was categorized as methylated (> = 40%), intermediate (20–40%), or unmethylated (< = 20%) based on the CG methylation level.

### ChIP-seq and DNase-seq data and alignments

In this study we utilized previously generated chromatin immunoprecipitation followed by high-throughput sequencing (ChIP-seq) data including H3K27me3 [31], H3K9ac [30] and H3K9me2 [12] along with novel H3K56ac data. Novel data was generated from B73 shoot tissue (described above) and ChIP-seq was performed following the general protocol of Zhang et al. [66]. Additionally, we utilized previously generated assay for accessible chromatin with high-throughput sequencing (DNase-seq) data [30]. Chromatin data was accessed from the Sequence Read Archive (SRA) database and can be retrieved through the accession numbers SRR5436222, SRX2527280, SRR1482362 and SRR5218002.

Adapter sequences were removed from raw reads using Trimmomatic version 0.33 [67] with default setting. Qualified reads were aligned to maize B73v4 genome using bowtie 1.1.1 [68] with the following parameters: -m 1 -v 2—best—strata—chunkmbs 1024 -S. Only uniquely mapped reads were retained and duplicated reads were then removed using rmdup module from samtools version 0.1.19 [65]. Output bam files were used to count the number of reads aligning to each 100bp window of the B73v4 genome. Counts were normalized per million mapped reads (each 100bp window count was divided by one million and then by the total count across the genome).

### Analysis of per-family TE chromatin modification patterns

The analysis of per-family DNA methylation (or other chromatin modifications) was restricted to the set of TEs within families containing > = 20 non-nested elements. Nested TEs are those elements with coordinates completely within another TE. There are 564 families in B73 and 401 that have at least 20 non-nested members in all four assessed genomes (S1 Table). Each 100bp window was assigned to the closest annotated TE using the bedtools closest function so that each window was only accounted for once and was only assigned to its closest TE. Although the orientation is generally known for LTR elements, it is rarely known for TIRs. In order to consistently plot trends surrounding TEs we compared the CG methylation levels in the 1kb flanks of each TE and then designate the flank with the higher methylation level to be plotted on the left (ie upstream of the TE). Averages were calculated by grouping TEs by their TE characterizations (order and family) and averaging within each 100bp window overlapping the TE body (normalized values) and within 1kb flanking upstream and downstream of the annotated sequence (actual distances). Relative distance was determined for the 100bp windows within the annotated TE (normalized to a 1kb window). Average CG and CHG methylation flanking TE families was used for k-means clustering for TIR and LTR plots separately using the kmeans function. The k-means clustering was performed using 2–5 clusters but visualization of the outputs suggested the presence of three distinct clusters and the classifications were performed using a k-means = 3 clustering. Heatmaps were ordered based on clusters and the 100bp windows overlapping a TE body were collapsed and averaged. Further comparisons of the defined clusters (H, M and L) were based on analyses of average values across all members. For DNA methylation, the context specific levels of DNA methylation from WGBS for each 100bp window across the genome were utilized. For chromatin modifications, the normalized counts per million (CPM) from ChIP-seq were calculated for each 100bp window and the average CPM across elements belonging to each category (H, M, or L) was determined. Determination of distance to genes was defined using bedtools closest with every TE being assigned to the single closest gene.

### TE Expression analysis

Per-family TE expression was previously summarized [32] for 23 tissues of B73 [33]. Expression for each family summarized in reads per million (RPM) was downloaded from https://github.com/SNAnderson/maizeTE_variation (file: Walley_TEfamily_expression_18Jan19.txt.gz). TE families were considered expressed if the RPM value exceeded one in at least one tissue. When assessing expressed TE families across tissues, values were calculated through a log2 normalization of the family level expression for each tissue sample.

### Analysis of methylation at TE absent sites and TE present flanks

The analysis of haplotypes with and without the TE was performed based on the set of site-defined polymorphisms identified for four maize genotypes [29]. In order to have a complete list of TE insertion sites data was merged across all pairwise comparisons with every defined site in an individual genome being maintained. CG methylation levels were determined on an individual genotype basis with alignment to the corresponding genome assembly. Insertion sites were based on the 100bp window overlapping the defined site in the haplotype absent of the TE (S9 Fig). Only sites with CG methylation data in this window were considered for analyses. Sites were then classified as methylated (> 40%), intermediate (20–40%), or unmethylated (<20%) based on the genome-wide distribution of CG methylation. When assessing family-based insertion patterns, the subset of 193 TE families with at least 10 insertions with DNA methylation data were considered (S4 Table). For haplotypes present for the TE, the flanking methylation was determined based on the 100bp windows on either side of the TE, but not overlapping the TE coordinates (S9 Fig). A single classification was made based on the average CG methylation for these flanking windows. When identifying family-based proportions of spreading, the subset of 150 TE families with at least 4 survey-able unmethylated insertion sites were considered (S4 Table).

## Supporting information

S1 Table Number of TEs and TE famiulies within each of the four genomes analyzed in this study. (XLSX)

S2 Table Summary information for whole genome bisulfite sequence data. (XLSX)

S3 Table Number of elements in each TE family in each of the four genomes. (XLSX)

S4 Table Classification of DNA methylation status for specific TE insertions and flanking regions. (XLSX)

S1 Fig The proportion of 100bp tiles with >2X coverage for WGBS data in B73_Shoot, PH207_Shoot, W22_leaf and Mo17_leaf (left to right) when mapped to the corresponding genome was determined. For each dataset/genome the proportion of 100bp tiles with >2x coverage for all regions (left), only genes (middle), and only TEs (right) is shown.
(TIF)

S2 Fig A WGBS dataset for maize genotypes (B73, W22, PH207, and Mo17) was mapped to the corresponding genome. (A) The level of DNA methylation in each sequence context (CG and CHG) was determined for each 100bp tile and histograms of CG and CHG DNA methylation are shown with classifications of methylated (purple), intermediate (orange), and unmethylated (green) regions indicated. B) All 100bp tiles were classified as TE, exon, intron, or intergenic based on B73v4 annotations. Each 100bp tile within these regions were classified into as methylated (> = 40%) intermediate (methylation levels > 20% and <40%) or unmethylated (methylation levels <20%). The proportion of 100bp tiles classified as methylated, intermediate or unmethylated in each type of annotation was determined.
(TIF)

S3 Fig The DNA methylation levels within and 1kb on either side of TIR and LTR elements in the maize genome was assessed. Each 100bp bin was assigned to the closest annotated TE in the B73v4 genome. The average DNA methylation levels for LTR (blue) and TIR (black) elements is shown in the CG (A), CHG (B) and CHH (C) contexts. Internal regions of TEs were normalized to a length of 1kb and are marked by vertical dotted lines.
(TIF)

S4 Fig Consistency of methylation profiles surrounding TEs throughout vegetative development. Per-family CG methylation profiles were determined as in Fig 2 using WGBS data from two additional tissues of B73 (B73 root and B73 leaf) and compared to the profiles for B73 shoot tissue. The heatmaps retain the same clustering order that was determined for B73 shoot data (as in Fig 2). Very similar consistent patterns are also observed using CHG methylation profiles.
(TIF)

S5 Fig Expression of TE families in three clusters defined as having high (H), moderate (M) or low (L) levels of CG and CHG methylation in flanking regions. The per-family expression level for each TE family was determined in a panel of 23 tissues of B73 using RNAseq data. The proportion of LTR (A) or TIR (B) families that were classified as high (H), moderate (M) or low (L) flanking methylation levels that have detectable (average RPM > 1) expression is shown. The number indicated above each bar represents the number of families expressed within each group. (C) For LTR TE families with detectable expression (average RPM > 1) a clustering was performed based on the log2 of the family level expression for that tissue sample. (D) A similar analysis is of TE expression is shown for TIR families.
(TIF)

S6 Fig A region on chromosome 1 of the B73v4 and W22 genomes is displayed as a schematic of genes (rectangles) and TEs (triangles). All TEs are labeled with their TE family. The blue TE is representative of a site-defined polymorphic TE where the insertion is in the W22 genome. The dashed red lines indicate the location of the empty site in B73. 100bp bins are represented with black dashed lines along the chromosome. These bins were used for analysis of methylation and chromatin data across genomes. The red line indicates the 100bp bin representative of the empty site while the purple and blue lines indicate the flanking 100bp bins of the empty site and TE, respectively.
(TIF)

S7 Fig Identification of excision events. The target site duplication (TSD) was identified for each empty site and the corresponding TE “insertion” was assessed for presence of the left and right TSD. If the sequence of both TSDs were identical to the insertion site, the event was classified as an insertion event (N = 4707). If neither TSD sequence matched, the TE was classified as a putative excision event (N = 579), and TEs with one matching TSD sequence were classified as unknown (N = 1158). The relative proportion of these groups identified as unmethylated, intermediate, and methylated was determined.
(TIF)

S8 Fig Consistency of DNA methylation changes near TEs. A subset of 613 TEs identified as absent in B73 (empty site) and present in W22, PH207, and Mo17 were identified. These included 88 examples in which the B73 empty site is unmethylated and 76 of these have flanking methylation gains in at least one other genotype. (A) The proportion of these 76 gains that are observed in 1, 2 or 3 genotypes was determined and plotted for TIRs and LTRs, respectively (A). (B) An example of a locus on chromosome 1 with an unmethylated empty site in B73 and gains of methylation in flanking regions for the other three genotypes is shown.
(TIF)

S9 Fig CG, CHG, and CHH DNA methylation profiles at LTR elements with or without spreading of DNA methylation. For these plots the orientation is based on the annotation of the element (rather than the level of DNA methylation in flanking regions). For elements that are inserted into unmethylated empty sites we assessed the DNA methylation profile based on the proportion of bins methylated (>40% for CG/CHG and >2% for CHH) for elements with spreading (purple) or without spreading (green) of DNA methylation in B73.
(TIF)

S10 Fig Analysis of attributes for members TEs classified as spreading or non-spreading. The proportion of superfamily designations for TEs classified as spreading or non-spreading. (A-B) For all elements classified as spreading or non-spreading the distance to the nearest gene was determined and binned overlapping, within 1kb, 1-5kb or >5kb. The proportion of elements with varying proximity to genes was compared for the spreading and non-spreading elements within TIRs and LTRs. LTR elements (C) are classified into copia (RLC), gypsy (RLG) or unclassified (RLX) superfamilies. TIR elements (D) are classified as hAT (DTA), CACTA (DTC), PifHarbinger (DTH), Mutator (DTM), Tc1/Mariner (DTT) or unclassified (DTX). (E-F) A similar analysis was done to compare the proportion of families with different copy numbers in spreading and non-spreading groups of LTR (E) or TIR (F) elements. Family size was classified into 4 groups. 20–50 members, 50–100 members, 100–200 members, and >200 members from light to dark color respectively. (G-H) The length of LTR and TIR elements was compared between the spreading and non-spreading groups. (I) For LTR elements the distribution of LTR similarities (% sequence identity) is shown for spreading and non-spreading elements. The greater % identity indicates younger age of the elements.
(TIF)

S11 Fig Analysis of CG density for spreading (purple) and non-spreading (green) for LTRs (left) and TIRs (right). The average number of cytosine sites per 100bp window for the 1kb flanking (top) and TE body (bottom) in each context (CG, CHG, and CHH) was calculated and the proportion of cytosines was plotted for each TE.
(TIF)
