# Supplementary figures and images for: Monitoring the interplay between transposable element families and DNA methylation in maize
Source: PLoS Genet. 2019 Sep 9;15(9):e1008291. doi: 10.1371/journal.pgen.1008291 (PMC6752859; doi:10.1371/journal.pgen.1008291)

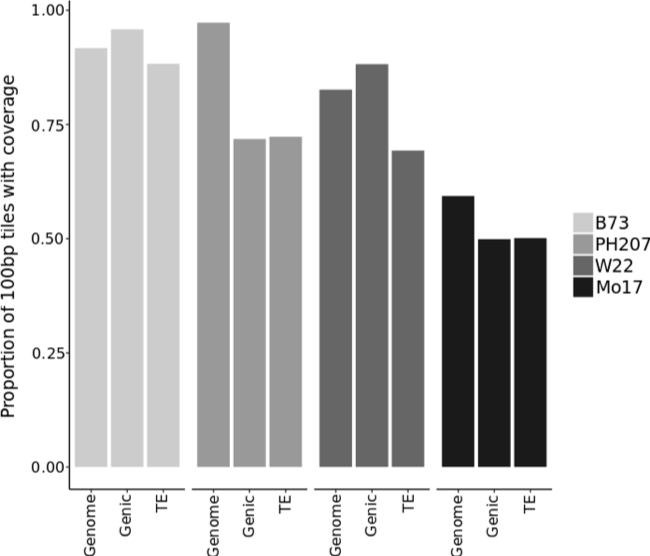

Supplement: S1 Fig — The proportion of 100bp tiles with >2X coverage for WGBS data in B73_Shoot, PH207_Shoot, W22_leaf and Mo17_leaf (left to right) when mapped to the corresponding genome was determined. For each dataset/genome the proportion of 100bp tiles with >2x coverage for all regions (left), only genes (middle), and only TEs (right) is shown. (TIF) [file pgen.1008291.s005.tif]

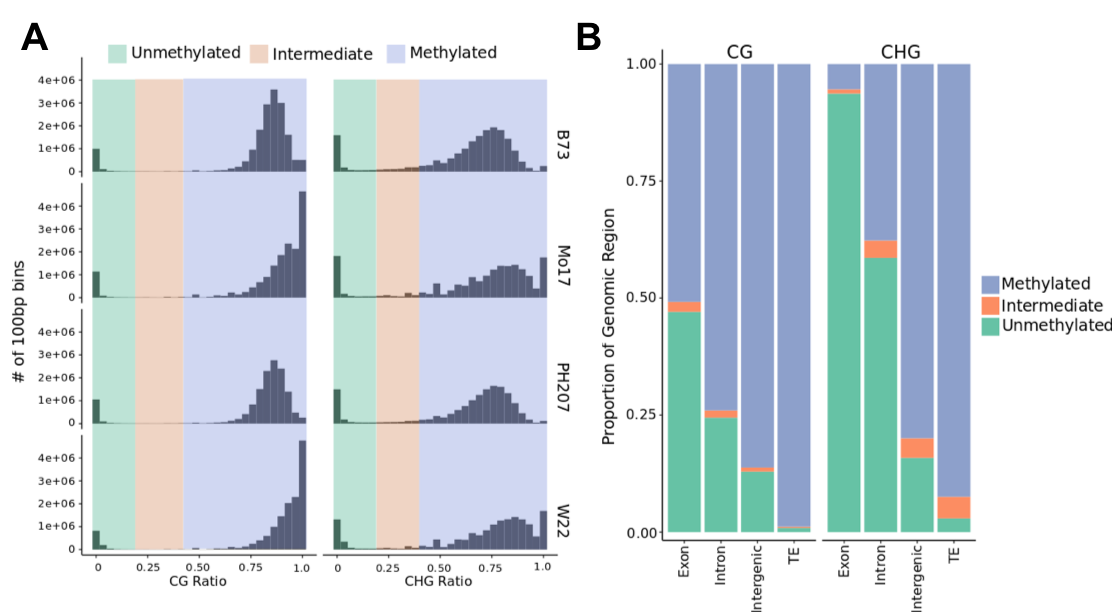

Supplement: S2 Fig — (A) The level of DNA methylation in each sequence context (CG and CHG) was determined for each 100bp tile and histograms of CG and CHG DNA methylation are shown with classifications of methylated (purple), intermediate (orange), and unmethylated (green) regions indicated. B) All 100bp tiles were classified as TE, exon, intron, or intergenic based on B73v4 annotations. Each 100bp tile within these regions were classified into as methylated (> = 40%) intermediate (methylation levels > 20% and <40%) or unmethylated (methylation levels <20%). The proportion of 100bp tiles classified as methylated, intermediate or unmethylated in each type of annotation was determined. (TIF) [file pgen.1008291.s006.tif]

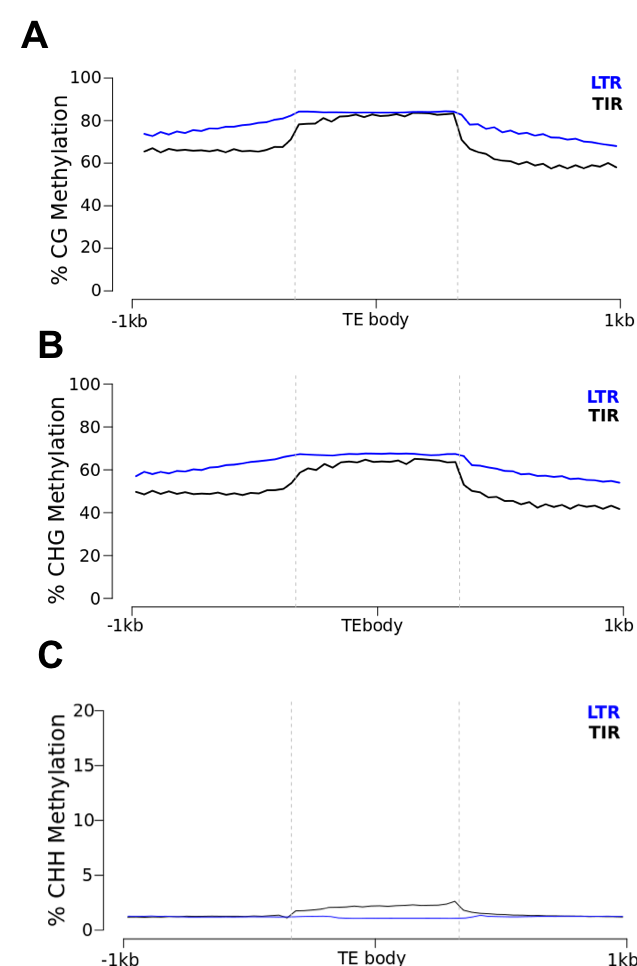

Supplement: S3 Fig — Each 100bp bin was assigned to the closest annotated TE in the B73v4 genome. The average DNA methylation levels for LTR (blue) and TIR (black) elements is shown in the CG (A), CHG (B) and CHH (C) contexts. Internal regions of TEs were normalized to a length of 1kb and are marked by vertical dotted lines. (TIF) [file pgen.1008291.s007.tif]

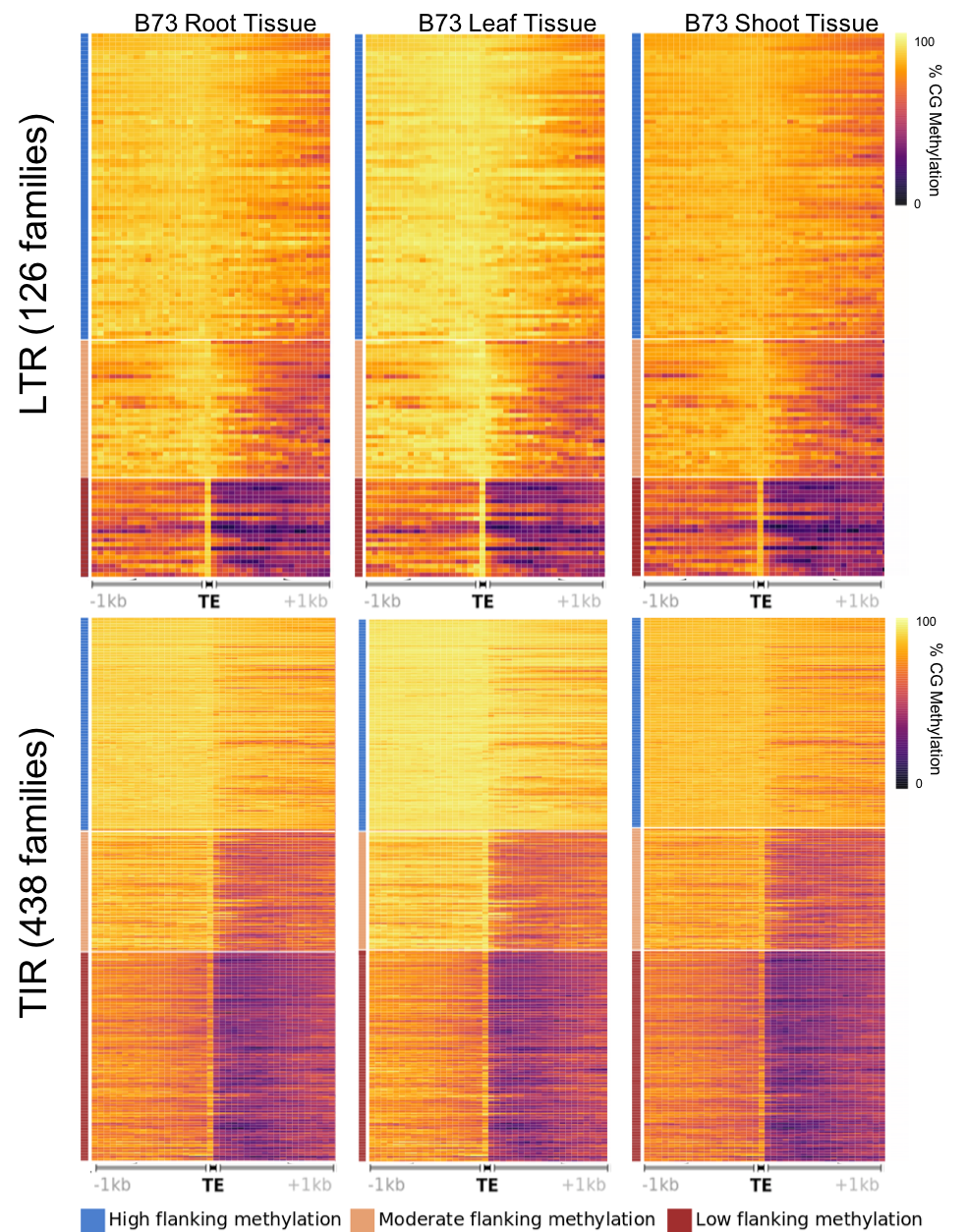

Supplement: S4 Fig — Per-family CG methylation profiles were determined as in Fig 2 using WGBS data from two additional tissues of B73 (B73 root and B73 leaf) and compared to the profiles for B73 shoot tissue. The heatmaps retain the same clustering order that was determined for B73 shoot data (as in Fig 2). Very similar consistent patterns are also observed using CHG methylation profiles. (TIF) [file pgen.1008291.s008.tif]

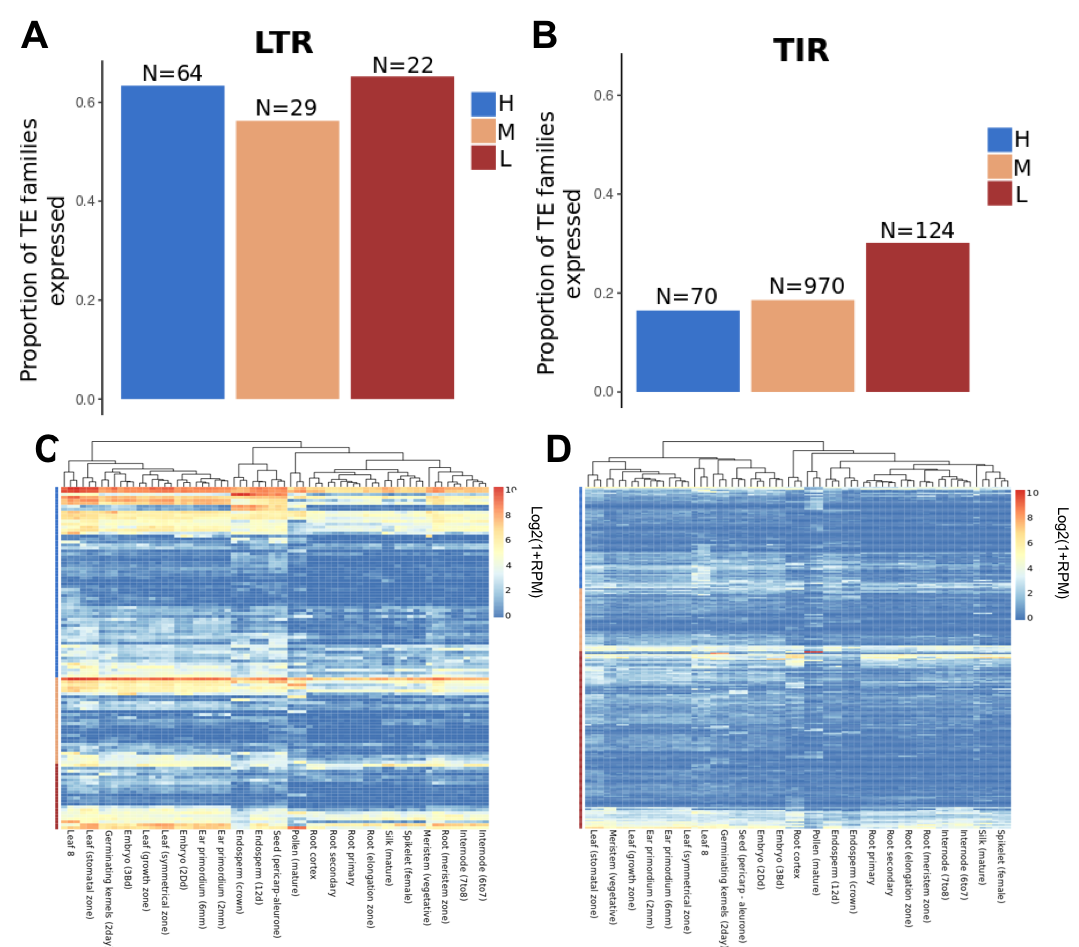

Supplement: S5 Fig — The per-family expression level for each TE family was determined in a panel of 23 tissues of B73 using RNAseq data. The proportion of LTR (A) or TIR (B) families that were classified as high (H), moderate (M) or low (L) flanking methylation levels that have detectable (average RPM > 1) expression is shown. The number indicated above each bar represents the number of families expressed within each group. (C) For LTR TE families with detectable expression (average RPM > 1) a clustering was performed based on the log2 of the family level expression for that tissue sample. (D) A similar analysis is of TE expression is shown for TIR families. (TIF) [file pgen.1008291.s009.tif]

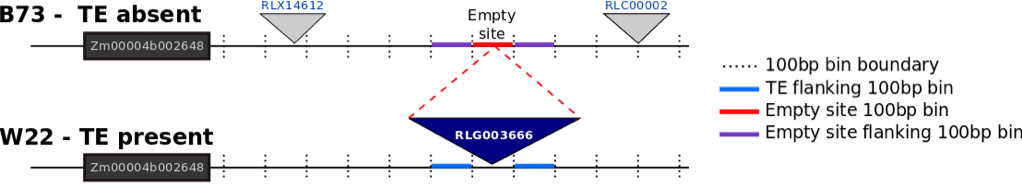

Supplement: S6 Fig — All TEs are labeled with their TE family. The blue TE is representative of a site-defined polymorphic TE where the insertion is in the W22 genome. The dashed red lines indicate the location of the empty site in B73. 100bp bins are represented with black dashed lines along the chromosome. These bins were used for analysis of methylation and chromatin data across genomes. The red line indicates the 100bp bin representative of the empty site while the purple and blue lines indicate the flanking 100bp bins of the empty site and TE, respectively. (TIF) [file pgen.1008291.s010.tif]

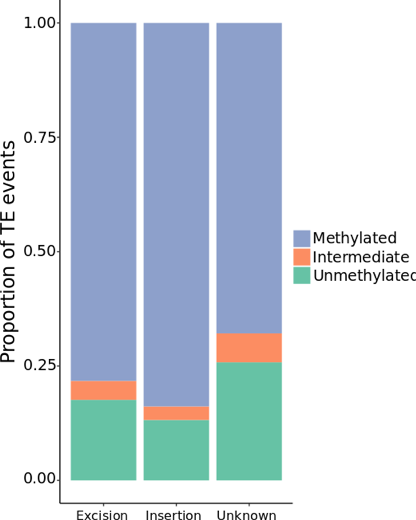

Supplement: S7 Fig — The target site duplication (TSD) was identified for each empty site and the corresponding TE “insertion” was assessed for presence of the left and right TSD. If the sequence of both TSDs were identical to the insertion site, the event was classified as an insertion event (N = 4707). If neither TSD sequence matched, the TE was classified as a putative excision event (N = 579), and TEs with one matching TSD sequence were classified as unknown (N = 1158). The relative proportion of these groups identified as unmethylated, intermediate, and methylated was determined. (TIF) [file pgen.1008291.s011.tif]

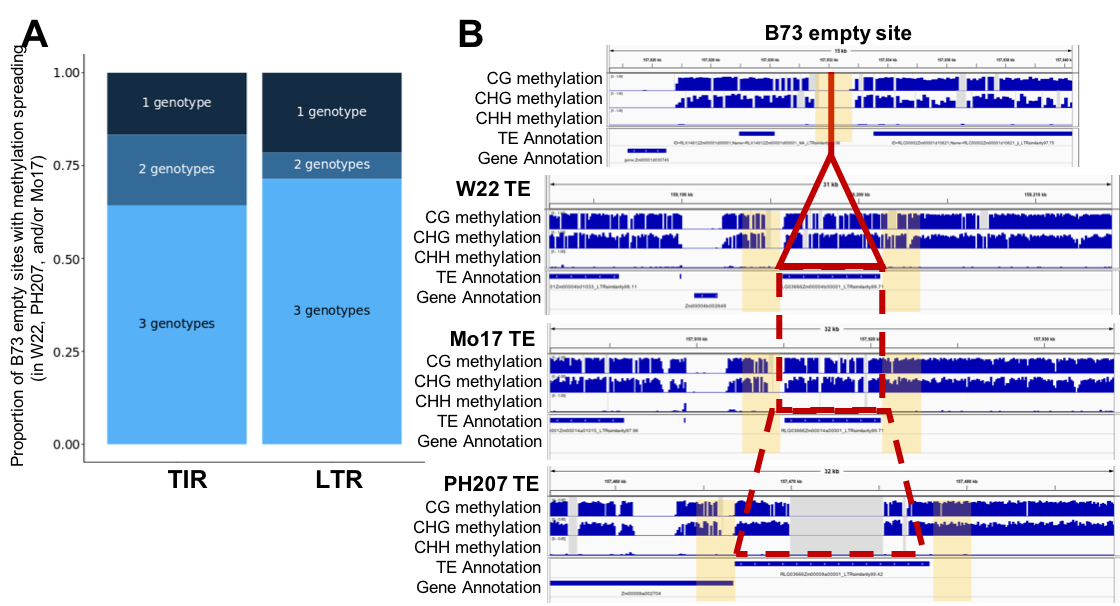

Supplement: S8 Fig — A subset of 613 TEs identified as absent in B73 (empty site) and present in W22, PH207, and Mo17 were identified. These included 88 examples in which the B73 empty site is unmethylated and 76 of these have flanking methylation gains in at least one other genotype. (A) The proportion of these 76 gains that are observed in 1, 2 or 3 genotypes was determined and plotted for TIRs and LTRs, respectively (A). (B) An example of a locus on chromosome 1 with an unmethylated empty site in B73 and gains of methylation in flanking regions for the other three genotypes is shown. (TIF) [file pgen.1008291.s012.tif]

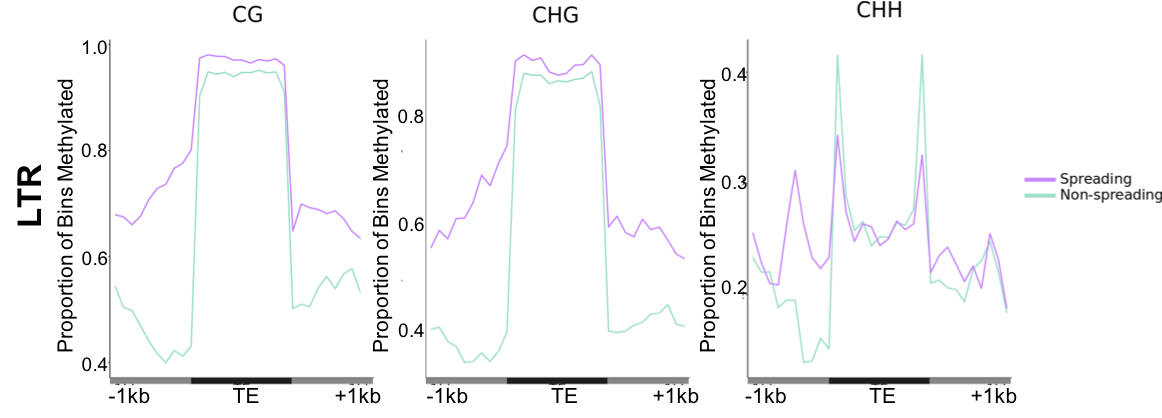

Supplement: S9 Fig — For these plots the orientation is based on the annotation of the element (rather than the level of DNA methylation in flanking regions). For elements that are inserted into unmethylated empty sites we assessed the DNA methylation profile based on the proportion of bins methylated (>40% for CG/CHG and >2% for CHH) for elements with spreading (purple) or without spreading (green) of DNA methylation in B73. (TIF) [file pgen.1008291.s013.tif]

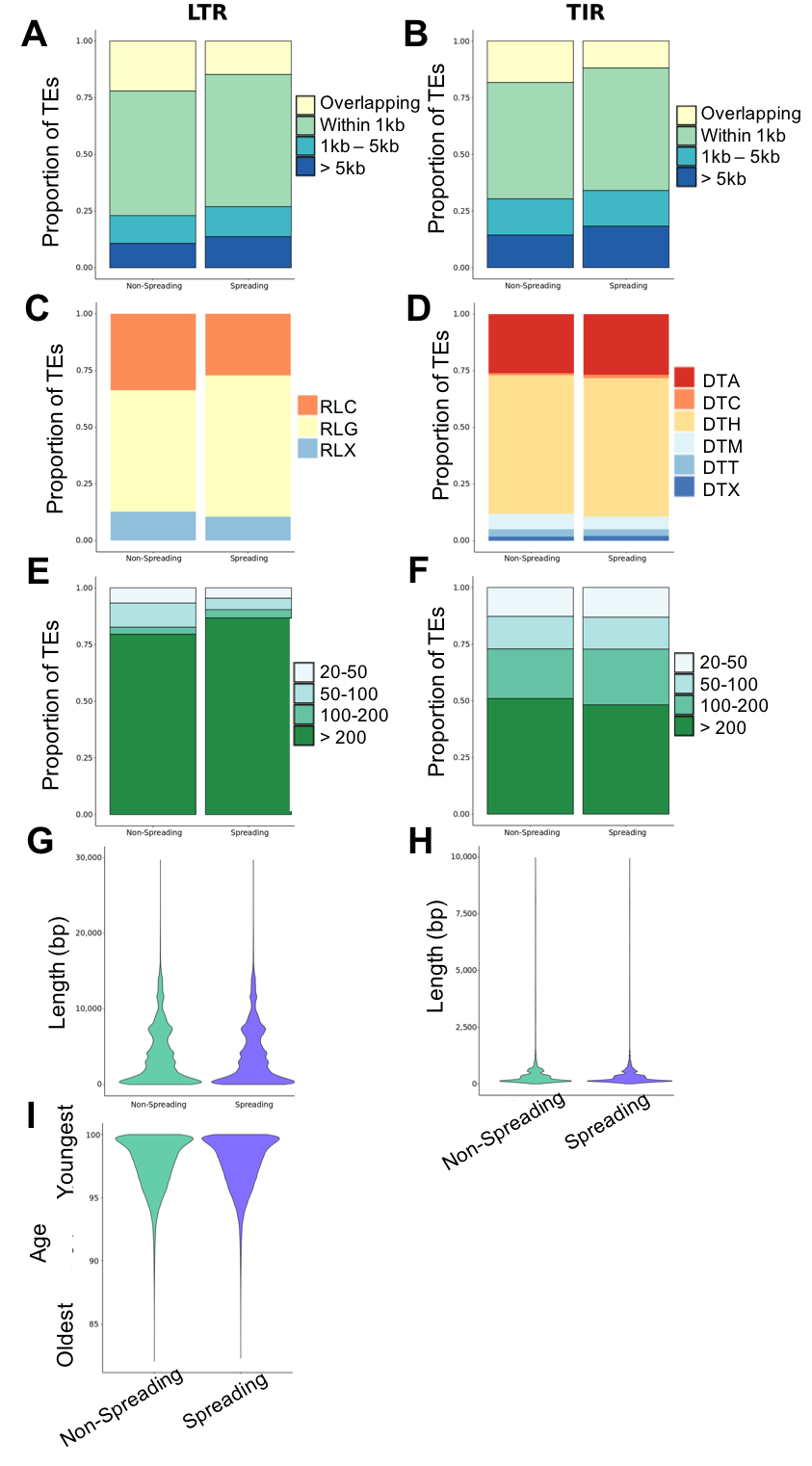

Supplement: S10 Fig — The proportion of superfamily designations for TEs classified as spreading or non-spreading. (A-B) For all elements classified as spreading or non-spreading the distance to the nearest gene was determined and binned overlapping, within 1kb, 1-5kb or >5kb. The proportion of elements with varying proximity to genes was compared for the spreading and non-spreading elements within TIRs and LTRs. LTR elements (C) are classified into copia (RLC), gypsy (RLG) or unclassified (RLX) superfamilies. TIR elements (D) are classified as hAT (DTA), CACTA (DTC), PifHarbinger (DTH), Mutator (DTM), Tc1/Mariner (DTT) or unclassified (DTX). (E-F) A similar analysis was done to compare the proportion of families with different copy numbers in spreading and non-spreading groups of LTR (E) or TIR (F) elements. Family size was classified into 4 groups. 20–50 members, 50–100 members, 100–200 members, and >200 members from light to dark color respectively. (G-H) The length of LTR and TIR elements was compared between the spreading and non-spreading groups. (I) For LTR elements the distribution of LTR similarities (% sequence identity) is shown for spreading and non-spreading elements. The greater % identity indicates younger age of the elements. (TIF) [file pgen.1008291.s014.tif]

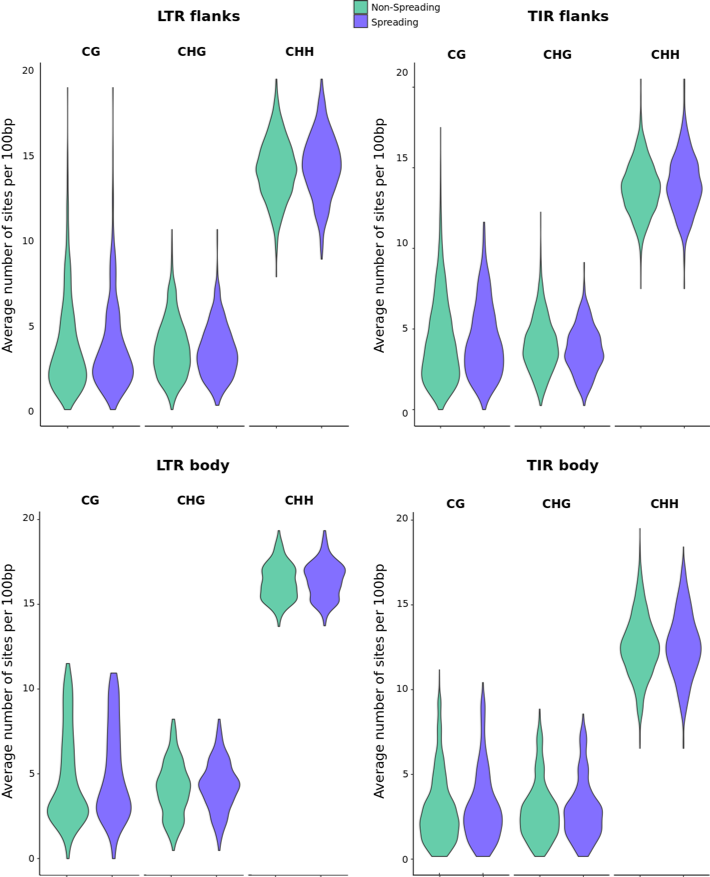

Supplement: S11 Fig — Analysis of CG density for spreading (purple) and non-spreading (green) for LTRs (left) and TIRs (right). The average number of cytosine sites per 100bp window for the 1kb flanking (top) and TE body (bottom) in each context (CG, CHG, and CHH) was calculated and the proportion of cytosines was plotted for each TE. (TIF) [file pgen.1008291.s015.tif]
